# Unexpected structures formed by the kinase RET C634R mutant extracellular domain suggest potential oncogenic mechanisms in MEN2A

**DOI:** 10.1016/j.jbc.2022.102380

**Published:** 2022-08-17

**Authors:** Yixin Liu, Orquidea De Castro Ribeiro, Outi Haapanen, Gregory B. Craven, Vivek Sharma, Stephen P. Muench, Adrian Goldman

**Affiliations:** 1 Molecular and Integrative Biosciences, Faculty of Biological and Environmental Sciences, University of Helsinki, Helsinki, Finland; 2 Department of Physics, University of Helsinki, Helsinki, Finland; 3 School of Biomedical Sciences, Faculty of Biological Sciences & Astbury Centre for Structural and Molecular Biology, University of Leeds, Leeds, United Kingdom; 4 HiLIFE Institute of Biotechnology, University of Helsinki, Helsinki, Finland

**Keywords:** RET, MEN2A, RET C634R mutant, membrane protein, protein-protein interaction, GDF15, GFRAL, GDNF, GFRα1, BN, Blue Native, CLD, cadherin-like domain, CRD, cysteine-rich domain, CTF, contrast transfer function, ECD, extracellular domain, EM, electron microscopy, GDF15, growth and differentiation factor 15, GDNF, glial-cell line–derived neurotrophic factor, GFL, glial-cell line–derived neurotrophic factor ligand, GFRα, GDNF receptor α, GFRAL, GDNF receptor α-like, MEN2A, multiple endocrine neoplasia type 2A, MD, molecular dynamics, NS-EM, negative stain-electron microscopy, SEC, size-exclusion chromatography, SEC-MALS, size exclusion chromatography-coupled multiangle static laser light scattering, WT, wild type

## Abstract

The RET receptor tyrosine kinase plays a pivotal role in cell survival, proliferation, and differentiation, and its abnormal activation leads to cancers through receptor fusions or point mutations. Mutations that disrupt the disulfide network in the extracellular domain (ECD) of RET drive multiple endocrine neoplasia type 2A (MEN2A), a hereditary syndrome associated with the development of thyroid cancers. However, structural details of how specific mutations affect RET are unclear. Here, we present the first structural insights into the ECD of the RET(C634R) mutant, the most common mutation in MEN2A. Using electron microscopy, we demonstrate that the C634R mutation causes ligand-independent dimerization of the RET ECD, revealing an unusual tail-to-tail conformation that is distinct from the ligand-induced signaling dimer of WT RET. Additionally, we show that the RET^C634R^ ECD dimer can form complexes with at least two of the canonical RET ligands and that these complexes form very different structures than WT RET ECD upon ligand binding. In conclusion, this structural analysis of cysteine-mutant RET ECD suggests a potential key mechanism of cancer induction in MEN2A, both in the absence and presence of its native ligands, and may offer new targets for therapeutic intervention.

Multiple endocrine neoplasia type 2A (MEN2A) is a hereditary polyglandular cancer syndrome that is typically characterized by childhood development of medullary thyroid carcinoma with a high probability of additionally suffering parathyroid and adrenal cancers. Genetically, MEN2A syndromes are caused by pathogenic germline RET variants, the most common of which contain activating mutations in one of six cysteine codons in the extracellular domain (codons 609, 611, 618, 620, 630, and 634 account for more than 98% of MEN2A cases) (1, 2, 3). Among these, the C634F/G/R/S/W/Y mutations are both the most frequently identified and the most aggressive, being designated high risk by the American Thyroid Association (4). Historically, these cancers have been associated with poor prognoses, developing and progressing throughout childhood, and have had limited treatment options. In 2020, the first highly RET-selective kinase inhibitor (Selpercatinib) was approved for the treatment of RET-mutant medullary thyroid carcinoma, showing marked and durable antitumor activity (5, 6). Cysteine-mutant RET now represents a clinically validated drug target, and a comprehensive understanding of the molecular mechanisms of cysteine-mutant oncogenic activation of RET is required to aid in diagnosis and treatment.

The extracellular domain (ECD) of RET comprises four cadherin-like domains (CLDs) and a cysteine-rich domain (CRD) that collectively coordinate ligand recognition and subsequent signal transduction. A variety of ligands have been identified to signal through RET, including the four glial-cell line–derived neurotrophic factor ligands (GFLs), glial-cell line–derived neurotrophic factor (GDNF), neurturin (NTRN), artemin (ARTN), and persephin (PSPN) (7, 8, 9, 10, 11), and the structurally related growth and differentiation factor 15 (GDF15) (12, 13, 14, 15). These RET ligands exist as soluble homodimers and their binding to RET is mediated through pairing with specific membrane-bound coreceptors, the expression of which is tissue dependent. Under normal physiology, activation occurs sequentially: the dimeric GFLs or GDF15 first recruit two copies of the GDNF receptors (GDNF α GFRα1-4) or GDNF receptor α-like (GFRAL) and this complex is further coordinated by two copies of RET, yielding a 2:2:2 hexameric tripartite complex that exhibits a “butterfly” conformation (16). Different ligand/coreceptor pairs result in distinct signaling outcomes: for example, the activation of the RET/GDNF/GFRα1 signaling pathway is critically involved in the maintenance of dopaminergic neurons (17), while elevated RET/GDF15/GFRAL signaling suppresses energy intake and is associated with the cancer cachexia syndrome (18). Although the ligands all activate intracellular signaling through dimerization of RET, there are substantial structural and conformational differences of the ligand, coreceptor, and RET in their complexes. Notably, the angles between the two “wings” of the RET complexes are approximately 60°, 105°, 108°, and 125° for the RET/GDF15/GFRAL, RET/NTRN/GFRα2, RET/ARTN/GFRα3, and RET/GDNF/GFRα1 complexes, respectively (16). These extracellular structural differences impact the relative conformations of the intracellular kinase domain dimerization and result in differential signaling outcomes (19, 20).

The C634R mutation in RET^CRD^ induces RET dimerization *via* a C630-C630 intermolecular disulfide bond and is one of the most common mutations in MEN2A cases (3, 21). However, a structural understanding of how this dimerization leads to constitutive activation of the kinase domains has so far been lacking. Previous cellular studies have shown contradictory results regarding the response of RET^C634R^ to ligand stimulation, with downstream phosphorylation being either increased upon the addition of GDNF (22, 23) or unchanged (24). Therefore, it remains uncertain whether dimeric RET^C634R^ is able to bind to its ligands and coreceptors extracellularly. Furthermore, if RET^C634R^ does associate with its ligand and coreceptors, does the C630-C630 disulfide bond introduce conformational restrictions that prevent binding in the same 2:2:2 stoichiometry and “butterfly” conformation as the WT RET (RET^WT^)?

In this study, we investigate the function of the RET^C634R^ ECD oncogenic mutant and report electron microscopy (EM) models of both the dimeric RET^C634R^ ECD mutant and its complex with GDF15/GFRAL as well as a cryo-EM model of the WT RET/GDF15/GFRAL extracellular domain complex. We report the first structural investigation of the unliganded RET^C634R^ ECD dimer and show that the two RET^C634R^ protomers associate with each other through the CRDs with the N-terminal domains pointing outward. We also observe and describe a unique “twin-butterfly” conformation of GDF15/GFRAL binding to RET^C634R^ ECD in a mechanism that differs from that of the RET^WT^ complexes and that of RET^C634R^/GDNF/GFRα1, forming a dodecameric tripartite complex. The results suggest the existence of divergent oncogenic activation mechanisms of the RET^C634R^ mutant resulting from configurational changes of unliganded and liganded RET^C634R^ extracellular domain complexes. These findings were further supported by molecular dynamics (MD) simulations, which found that the RET^C634R^ ECD dimer can adopt a diverse range of activating conformations driven by aberrant disulfide stapling.

## Results

### Characterization of the extracellular domain of dimeric RET^C634R^

**Figure 1:**
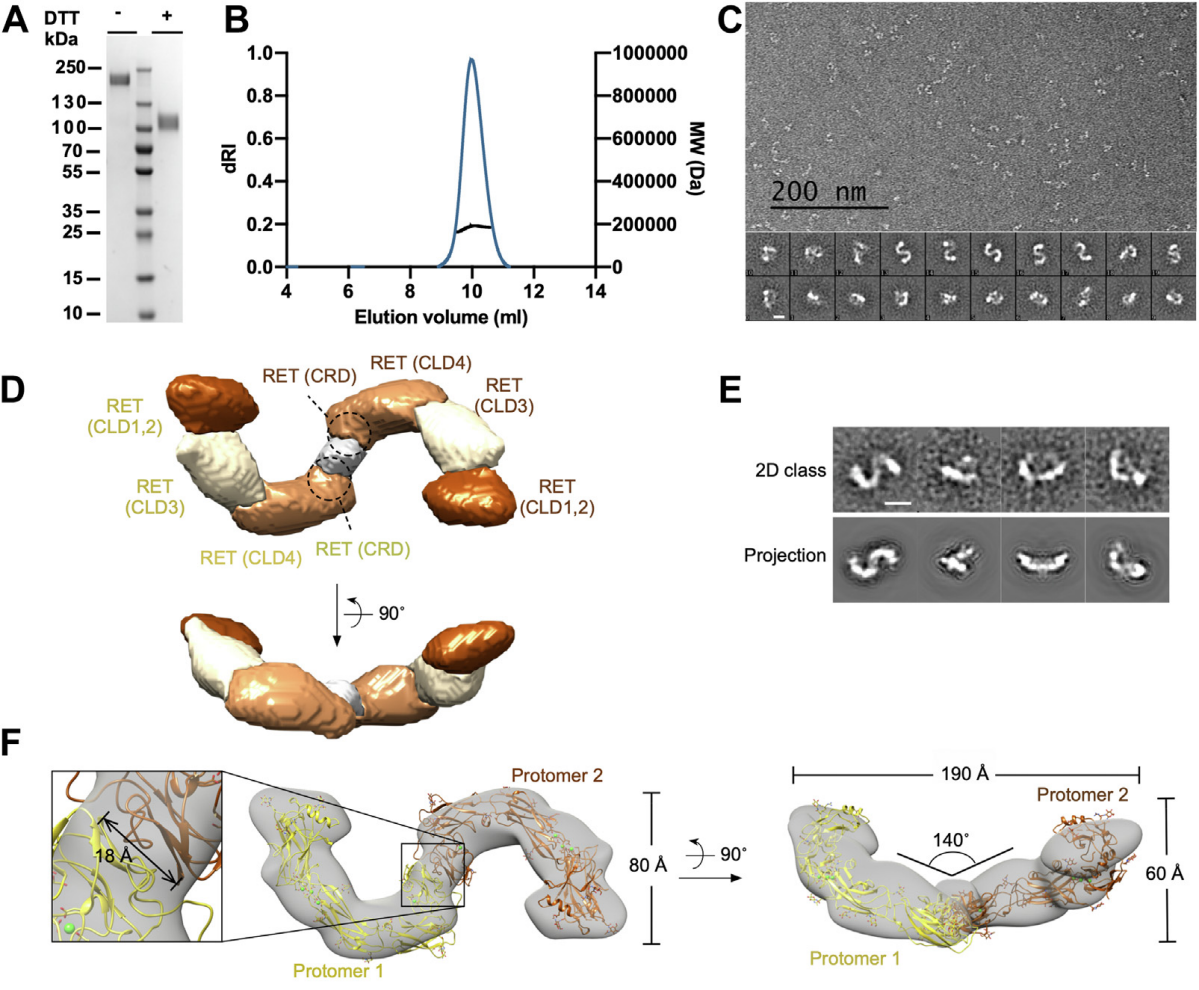
**Characterization of the extracellular domain of the RET**^**C634R**^**dimer.***A*, coomassie-stained SDS-PAGE image showing the dimeric RET^C634R^ mutant under reducing and nonreducing conditions. *B*, sample homogeneity assessed using SEC-MALS. *C*, a representative micrograph and 2D class averages of RET^C634R^. Scale bar for 2D class images represents 10 nm. *D*, *top* and front views of the segmented maps of the NS-EM model of RET^C634R^ dimer. The segmented maps are colored to highlight the CLD1/2s, CLD3s, CLD4s, and CRDs in *brown*, *off-white*, *orange*, and *gray*, respectively. *E*, comparison of representative 2D class averages and the projection images of the reconstructed NS-EM model. The scale bar represents 10 nm. *F*, *top* and front views of the NS-EM model of RET^C634R^ dimer docked with two RET^ECD^ (*yellow* and *brown*, modified from PDB ID: 6Q2J). An enlarged view of the density between the C-termini of RET^C634R^s is shown (*left side*, *boxed*), showing the distance between the two P622 residues in the docked structures. CLD, cadherin-like domain; CRD, cysteine-rich domain; EM, electron microscopy; ECD, extracellular domain; SEC, size-exclusion chromatography; SEC-MALS, size exclusion chromatography-coupled multiangle static laser light scattering.

The expression of correctly folded and functional human RET requires the use of mammalian expression systems and we previously reported that the addition of a cleavable C-terminal Fc tag facilitates the expression of RET^C634R^ ECD in its dimeric form, mediated by an intermolecular C630-C630 disulfide bond (25). For this study, we further optimized the expression construct (see Experimental procedures for details) and were able to isolate dimeric RET^C634R^ ECD in high homogeneity (Fig. 1, *A* and *B*). To investigate the structural configuration of dimeric RET^C634R^ ECD, we used single particle negative stain EM (NS-EM) to generate a three-dimensional model. With a total of 3490 particles, both the single particles and the two-dimensional (2D) class averages (Fig. 1*C*) showed a clear “S” shape, suggesting a rotational C2 symmetry of the model. Initial model building and further model refinement performed with C2 symmetry agreed well with the 2D classes (Fig. 1, *D* and *E*). The three-dimensional (3D) refinement of the model converged at 22 Å resolution. The dimensions of the reconstructed model of the dimeric RET^C634R^ are 60 Å × 190 Å × 80 Å and the shape resembles a nonplanar “S”, comprising two “C”-shaped RET^C634R^ protomers that are connected tail-to-tail at the CRDs *via* a C630-C630 disulfide linkage. Segmentation of the 3D map (26) resulted in seven components, representing 2 × CLD1/2, 2 × CLD3, 2 × CLD4 and joint density in the center for 2 × CRDs (Fig. 1*D*). We docked one copy of RET^ECD^ (modified from PDB ID: 6Q2J) into each half of the density map, which gave a good overall match of both size and shape and defined the location of the individual domains in the segmented maps (Fig. 1*F*). There are no regions of joint density other than the CRDs, indicating that none of the CLDs interact across the dimer interface. Because the C-terminal residues E623-R635 of RET^ECD^ are not resolved in the previously published structures (*i.e.*, PDB ID: 6Q2J) (16), we could not define the position of the C630-C630 disulfide bond precisely. However, the measured distance between the two P622 residues of each RET^C634R^ protomer is ∼18 Å, leaving enough space to accommodate the missing region in the docked structures.

### The extracellular domain of RET^C634R^ binds to its ligands through a novel mechanism

**Figure 2:**
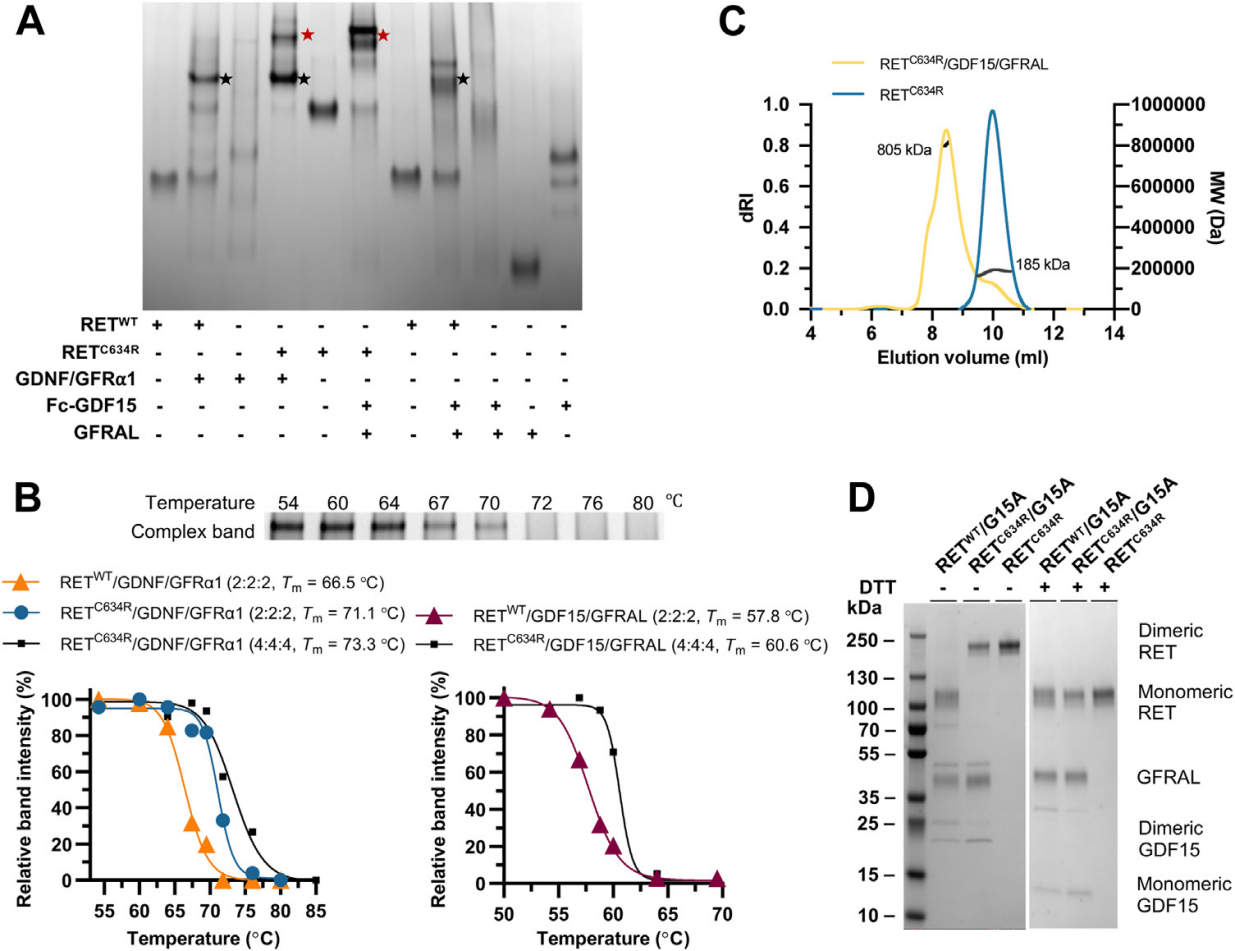
**Extracellular domain complex formation of RET/GDNF/GFRα1 and RET/GDF15/GFRAL.***A*, RET^C634R^ formed a higher order complex with GDNF/GFRα1 and GDF15/GFRAL (marked with *red stars*) compared with RET^WT^ in BN PAGE. The complexes of RET^WT^/GDNF/GFRα1, RET^C634R^/GDNF/GFRα1, and RET^WT^/GDF15/GFRAL with a molar ratio of 2:2:2 are marked with *black stars*. The Fc-GDF15 showed two major bands on the gel with the strongest band being the dimeric Fc_2_-GDF15 and the lower fainter band being the monomeric Fc-GDF15 as a result of Fc dissociation. *B*, stability measurement of the tripartite complexes of GDNF/GFRα1 (*left panel*) and GDF15/GFRAL (*right panel*) with RET^C634R^ and RET^WT^. Above: an example temperature challenge BN PAGE image of the 2:2:2 RET^WT^/GDNF/GFRα1 complex (please refer to Fig. S1 for full image). Band intensity of the complexes treated at various temperatures was quantified using ImageJ (43). Normalized band intensity was used to plot the melting curves, fitted by nonlinear regression to a sigmoidal model. *C*, characterization of RET^C634R^/GDF15/GFRAL (*yellow*), RET^C634R^ (*blue*) using SEC-MALS. The *right Y-axis* represents the calculated molecular weight (MW), while the *left Y-axis* is the differential refractive index (dRI). They are plotted against elution volume (X-axis). The calculated average molecular weights of the peaks are plotted (*black*), showing MWs of 185 kDa for RET^C634R^ dimer and 805 kDa for RET^C634R^/GDF15/GFRAL. *D*, coomassie-stained SDS-PAGE image showing purified RET^C634R^, RET^C634R^/GDF15/GFRAL, and RET^WT^/GDF15/GFRAL under reducing and nonreducing conditions. BN, Blue Native; GFRAL, GDNF receptor α-like; SEC-MALS, size exclusion chromatography-coupled multiangle static laser light scattering.

Dimeric RET^C634R^ is generally considered to be constitutively activated in the absence of ligand binding. However, previous studies have shown that RET^C634R^ signaling is further upregulated in respond to GDNF stimulation (22, 23), which may play a role in oncogenesis. It is currently unclear whether GDNF binds RET^C634R^ in the same manner as RET^WT^ and whether other GFL/coreceptor pairs share this activation capacity. In order to probe the mechanism of ligand-induced hyperactivation of RET^C634R^, we compared how RET^WT^ ECD and dimeric RET^C634R^ ECD bind two distinct ligand/coreceptor pairs (GDNF/GFRα1 and GDF15/GFRAL) using Blue Native (BN) PAGE. As expected, we found that RET^WT^ forms a stable 2:2:2 tripartite complex with GDNF/GFRα1, as indicated by the formation of a major band that migrates more slowly than any of its components (Fig. 2*A*). Interestingly, we found that dimeric RET^C634R^ also complexes with GDNF/GFRα1 to produce a major band that migrates similarly, suggesting that it too assembles predominantly as a 2:2:2 tripartite complex. However, RET^C634R^/GDNF/GFRα1 *also* forms a band with an even slower migratory shift, which is probably a higher-order complex (red star, lane 4). The RET^WT^/GDNF/GFRα1 mixture contains a substantial amount of uncomplexed component proteins unlike the RET^C634R^/GDNF/GFRα1 mixture, suggesting that the RET^C634R^/GDNF/GFRα1 complex, especially 2:2:2, may be more favored. In support of this, we found that both the 2:2:2 and higher-order RET^C634R^/GDNF/GFRα1 complexes are more thermally stable than the RET^WT^/GDNF/GFRα1 complex (Fig. 2*B*, left panel and Fig. S1), indicating that the C630-C630 disulfide bond has a stabilizing effect.

RET^WT^ forms a major complex with GDF15/GFRAL that migrates similarly to RET^WT^/GDNF/GFRα1, which is consistent in that both complexes are known to share a 2:2:2 tripartite composition. Surprisingly, however, we found that RET^C634R^/GDF15/GFRAL forms one major higher-order complex that migrates substantially more slowly than the RET^WT^/GDF15/GFRAL complex but similarly to the weaker band in the RET^C634R^/GDNF/GFRα1 sample (Fig. 2*A*). We found that this higher-order complex is moderately thermally stabilized (T_m_ = 60.6 °C) relative to the WT complex (T_m_ = 57.8 °C) (Fig. 2*B*, right panel). These results suggest that dimeric RET^C634R^ may be conformationally restricted from forming the 2:2:2 complex with GDF15/GFRAL but not with GDNF/GFRα1 or that the higher-order species is more favored in the case of GDF15/GFRAL.

To characterize these higher-order RET^C634R^ complexes further, we investigated the oligomeric state of RET^C634R^/GDF15/GFRAL using size-exclusion chromatography-coupled multiangle static laser light scattering (SEC-MALS). Analysis of the RET^C634R^/GDF15/GFRAL complex gave a calculated molecular weight of 805 kDa (Fig. 2*C*), approximately twice the size of the heterohexameric RET^WT^/GDF15/GFRAL (395 kDa, Fig. S2). To investigate whether the RET^WT^- and RET^C634R^-ligand complexes have the same molar ratio of RET:GDF15:GFRAL, we compared the composition of the individual subunits using nonreducing and reducing SDS-PAGE (Fig. 2*D*). Under reducing conditions, the RET^C634R^ mutant runs at the same molecular weight as RET^WT^, demonstrating the reduction of the C630-C630 disulfide bond. Indeed, we found that the RET^WT^- and RET^C634R^-complexes have the same molar ratio of all three components under reducing conditions. Taken together with the BN PAGE and SEC-MALS data, these results suggest that RET^C634R^ ECD forms a complex with GDF15 and GFRAL in a stoichiometry of 4:4:4 that must exist in a different configuration from the RET^WT^ ECD complex. Indeed, we measured that the affinity of RET^C634R^ ECD for GDF15/GFRAL (*K*_d_ = 8.5 μM) is actually weaker than that of RET^WT^ ECD (*K*_d_ = 3.2 μM) (25) (Fig. S3). We had initially anticipated that the dimeric nature of RET^C634R^ might offer an entropic advantage compared to monomeric RET^WT^ in forming a 2:2:2 hexameric ligand complex and that this would instead result in a stronger binding affinity. We inferred from this loss of affinity that dimeric RET^C634R^ is likely to be geometrically restricted from binding the ligands in the same conformation as two independent copies of monomeric RET^WT^, supporting the hypothesis that RET^C634R^ forms a complex with GDF15/GFRAL through a novel mechanism.

### Structural comparison of the RET^WT^ and RET^C634R^ extracellular domain complexes

**Figure 3:**
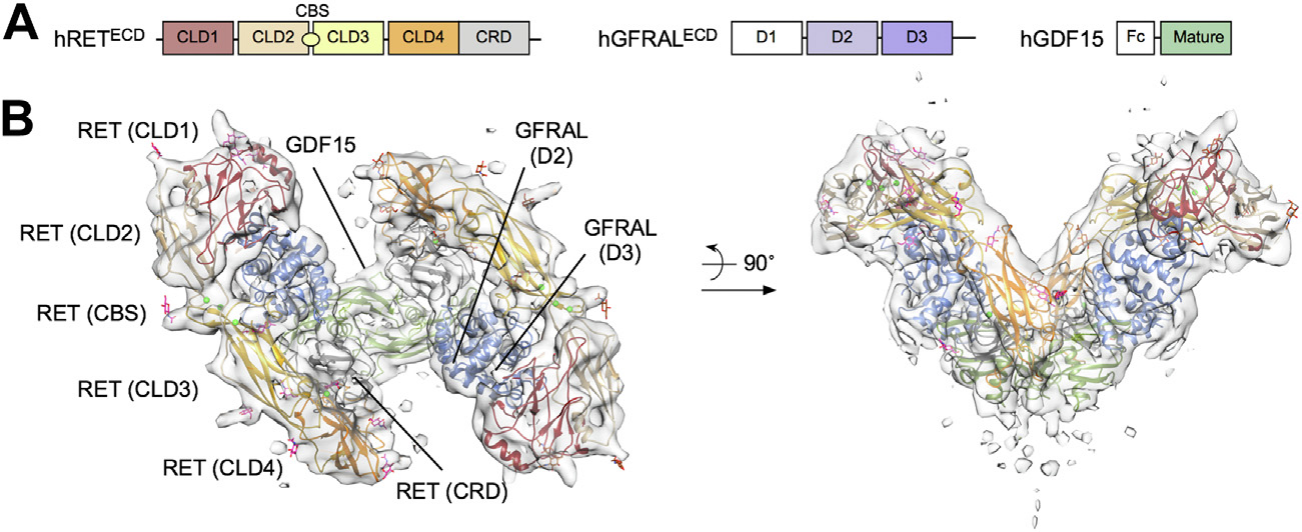
**Cryo-EM 3D model of the extracellular domain complex of RET**^**WT**^**/GDF15/GFRAL.***A*, schematic of hRET^ECD^, hGFRAL^ECD^, and Fc-tagged mature hGDF15 used in this study. *B*, a modified structure of RET^WT^/GDF15/GFRAL (PDB ID: 6Q2J) is fitted in the density. One N-acetylglucosamine (GlcNAc) is placed at each glycosylation sites to fit the density using Coot. The protein domains are colored according to the color scheme in (*A*). ECD, extracellular domain; GFRAL, GDNF receptor α-like; EM, electron microscopy.

In order to compare the character of the RET^WT^ and RET^C634R^ ligand complexes, we used EM to interrogate their structures. Accordingly, we first reconstituted the RET^WT^/GDF15/GFRAL complex and analyzed it by cryo-EM. We performed 3D model reconstruction with 142,083 particles of RET^WT^/GDF15/GFRAL using an initial model built from the 2D class averages, and the resulting refined model had an overall resolution of 8 Å (Fig. 3). Comparing our map to a previously published cryo-EM structure of the same complex (PDB ID: 6Q2J) (16), we observed only minor differences, most notably extra density, consistent with the additional glycosylation at certain sites. RET^ECD^ is heavily glycosylated, containing 11 glycosylation sites that influence the structure and function of RET (25, 27, 28). In our study, we used HEK293T cells to produce RET^ECD^ bearing near-native glycans and, at this resolution, densities of the base glycans at all the glycosylation sites could be observed (Fig. S4). Overall, two of the glycosylation site densities point toward the inside of the complex (N361 and N336) while the rest point outward.

**Figure 4:**
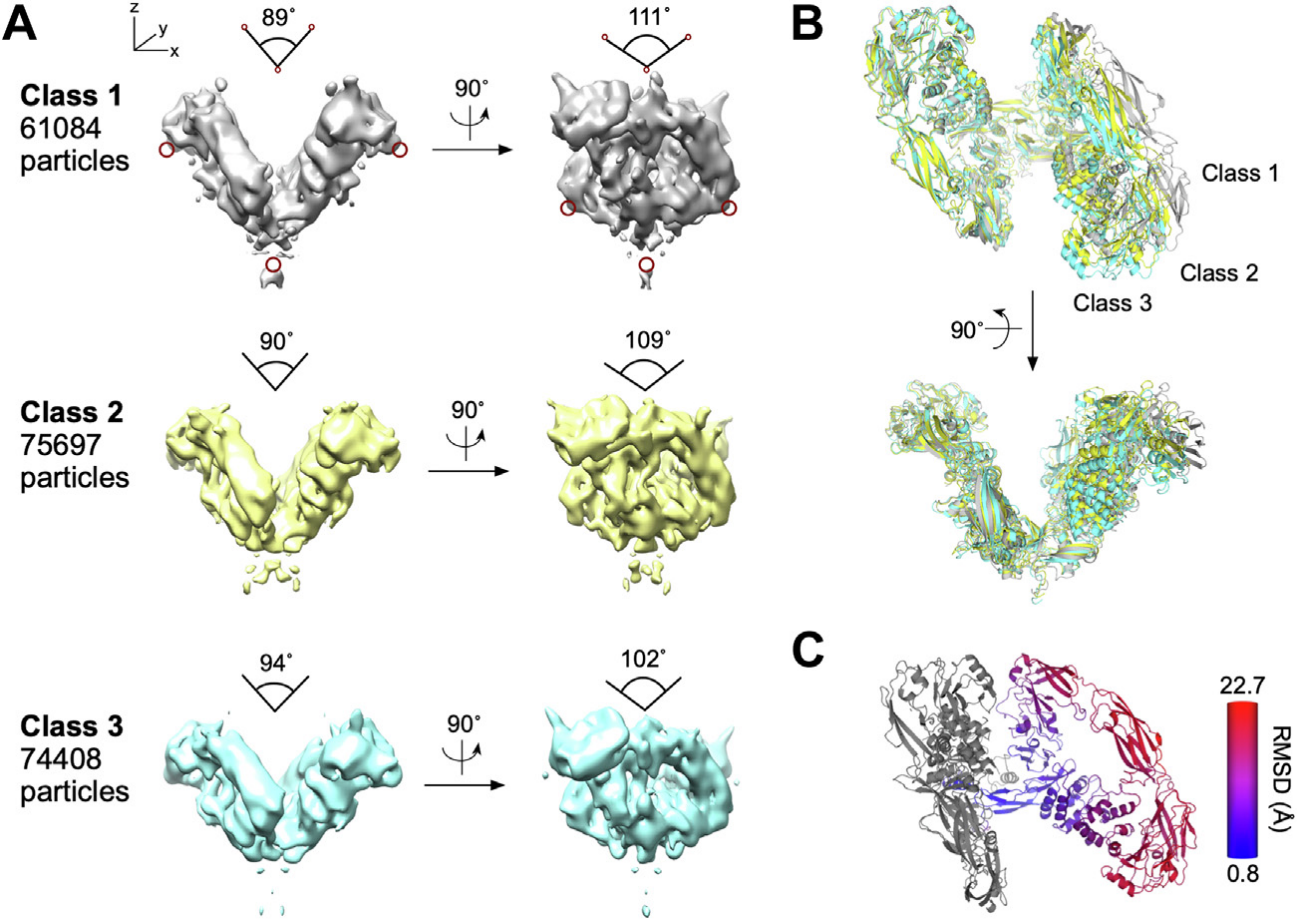
**Conformational variations of the extracellular domain complex of RET**^**WT**^**/GDF15/GFRAL.***A*, cryo-EM models auto-refined from three classes of particles. The three models share the same configuration but vary in the angles between the two “wings” of the complex along the x (89° to 94°) and y (102° to 111°) axes. The angles were measured using the positions marked by the *red circles*. *B*, comparison of the Flex-EM modeled structures of RET^WT^/GDF15/GFRAL after aligning one half of the complexes. Modeled structures for class 1, 2, and 3 are colored *gray*, *yellow*, and *cyan*, respectively. *C*, analysis of the conformational change of class 3 relative to class 1 as defined by the RMSD value after alignment of half of the complex (region colored in *gray*). EM, electron microscopy; GFRAL, GDNF receptor α-like.

In parallel, using the same data set, a 30-Å map was generated from the previously published cryo-EM structure of RET^WT^/GDF15/GFRAL (PDB ID: 6Q2J) that was low-pass filtered to 60 Å and used as a reference map for subsequent 3D classification and auto-refinement. This refined map also has an overall resolution of 8 Å and is in close structural agreement with the *de novo* model that we reconstructed using the RELION-built initial model as reference map. Analysis of three 3D classes generated during the reconstruction revealed movement of the two “wings” of the RET^WT^/GDF15/GFRAL complex (Fig. 4). Class 1 showed the narrowest (89°) and widest (111°) angles along the x and y axis, respectively, while class 3 had the widest (94°) and narrowest (102°) angles. Class 2 shared similar angles with class 1 along the x (90°) and y axes (109°). To assess these changes of conformation, the WT complex structure was fitted individually into the three refined cryo-EM maps from 3D classification using rigid-body flexible fitting. The resulting structures were then superimposed along one half of the complex for structural comparison. Overall, RET^ECD^ showed the highest degree of conformational change relative to GDF15 and GFRAL (Fig. 4*B*). Morphing of the structures suggests that the two “wings” of the complex are flexible, showing an “out-in” bend and “front-back” twist movement (Movie S1).

**Figure 5:**
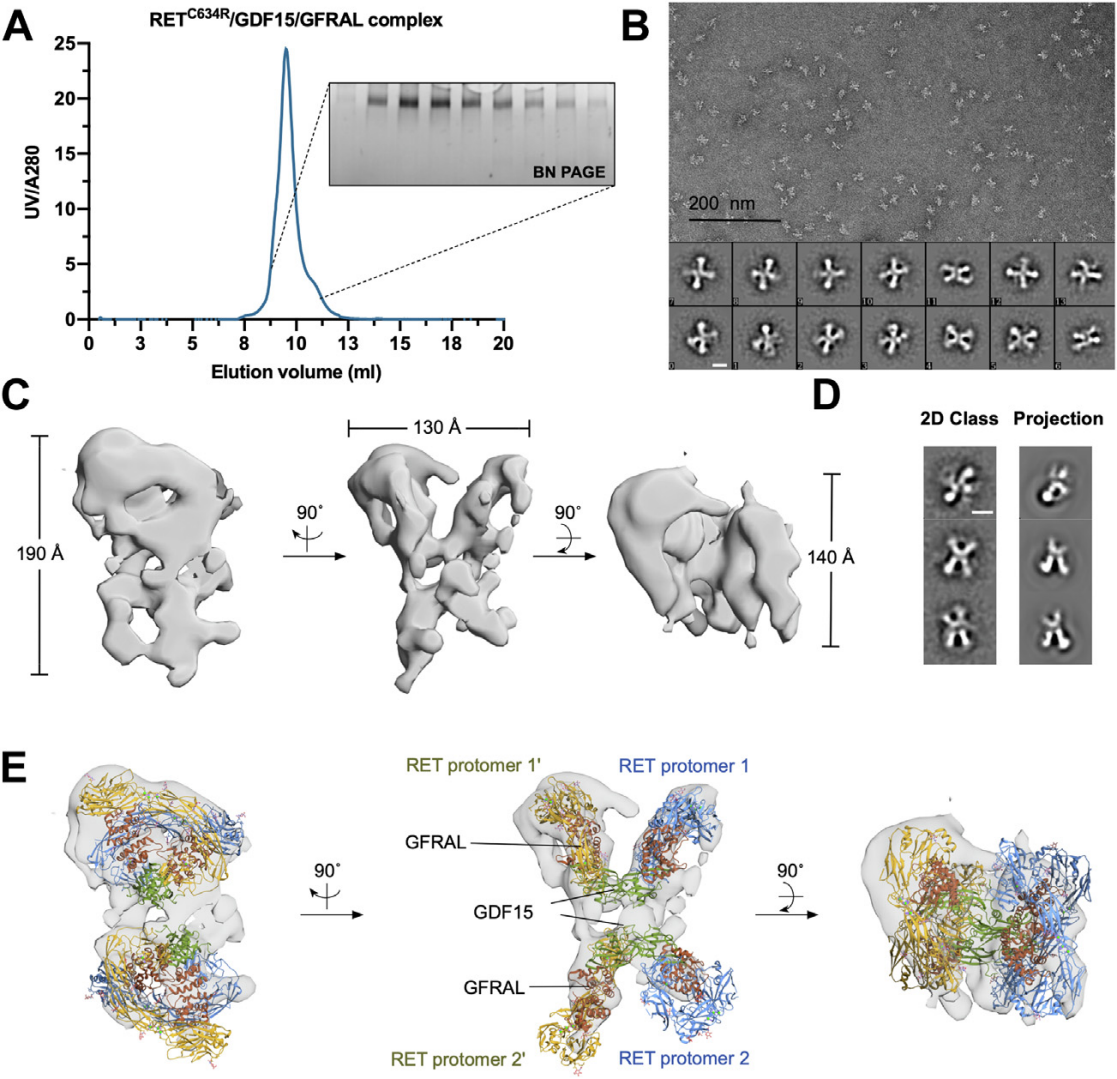
**Structural analysis of the extracellular domain complex of RET**^**C634R**^**/GDF15/GFRAL using negative stain EM.***A*, analysis of RET^C634R^/GDF15/GFRAL by SEC on a Superdex 200 10/300 increase column and BN PAGE. *B*, negative-stained particles from a representative field of the micrographs taken at 50,000 magnification and representative 2D class averages processed using EMAN2.3 with 11278 particles, presenting different views of the complex. Scale bar for 2D class images represents 10 nm. *C*, different views of the NS-EM model of RET^C634R^/GDF15/GFRAL with measured dimensions. *D*, comparison of representative 2D class averages and the projection images of the reconstructed NS-EM model. Scale bar represents 10 nm. *E*, side, front, and *top* views of the NS-EM model of RET^C634R^/GDF15/GFRAL docked with two RET^WT^/GDF15/GFRAL complexes (modified from PDB ID: 6Q2J). RET or RET′ protomers that belong to the same RET^C634R^ cross-linked dimer have the same color (*blue* or *yellow*). GFRAL and GDF15 are colored *brown* and *green*, respectively. BN, Blue Native; EM, electron microscopy; GFRAL, GDNF receptor α-like; SEC, size- exclusion chromatography.

The RET^C634R^/GDF15/GFRAL complex was reconstituted similarly to the RET^WT^ complex and purified by gel filtration (Fig. 5*A*). Analysis of the RET^C634R^/GDF15/GFRAL complex by negative stain EM showed that the particles were monodisperse with a distinct change in morphology with both single particles and the resultant 2D class averages showing an overall “X” shape with apparent two-fold rotational symmetry (Fig. 5*B*). An initial model built without symmetry (Fig. S5) agreed well with the overall “X” shape observed in the 2D classes and was roughly twice the size of the RET^WT^ complex. Further refinement was performed with no symmetry assigned (Fig. 5*C*). The dimensions of the reconstructed model of the RET^C634R^/GDF15/GFRAL complex are 190 Å × 130 Å × 140 Å, showing an “X” shape as the front view. Reprojections of the density match well with the 2D class averages, supporting the quality of the reconstructed model (Fig. 5*D*). In agreement with our hypothesis, two copies of the RET^WT^/GDF15/GFRAL structure could be docked back-to-back into the RET^C634R^/GDF15/GFRAL density map (Fig. 5*E*), confirming our observations in the biochemical and biophysical assays (Fig. 2). In particular, the top half of the density provides a much better fit for the complex than the lower half, with extra densities observed in the top half, which could be from Domain 1 (D1) in GFRAL that was not previously resolved. This conformational heterogeneity likely also explains the poorer resolution in the lower half. Given that the RET^WT^/GDF15/GFRAL complex has 2:2:2 stoichiometry and possesses C2 symmetry, we hypothesized that the RET^C634R^/GDF15/GFRAL complex, which has 4:4:4 stoichiometry and an “X” shaped 2D projection, would have D2 symmetry. However, assigning C2 or D2 symmetry in further refinements did not improve the resolution of the map (data not shown), likely due to conformational flexibility between the domains. At this resolution, we were able to assign the overall configuration of the complex with moderate confidence but were unable to model the disulfide linked loops fully. Since the two RET^C634R^ protomers in a RET^C634R^ dimer are linked by a disulfide bond and favor the formation of a 4:4:4 complex, it is likely that a pair of disulfide-linked RET^C634R^ dimers form two semi-independent 2:2:2 complexes *in trans* (Fig. 5*E*). Although the disulfide linkage itself cannot be resolved at this resolution, the distance between the P622 residues in the two RET^C634R^ protomers is ∼40 Å, which is longer than that of the unliganded RET^C634R^ dimer but could be spanned by residues E623-R635 that are missing from the model and known to be flexible (Fig. 1*F*). This suggests that ligand binding drives a conformational change of the C-termini of the ECDs of dimeric RET^C634R^, separating them from each other to accommodate the second heterohexamer.

**Figure 6:**
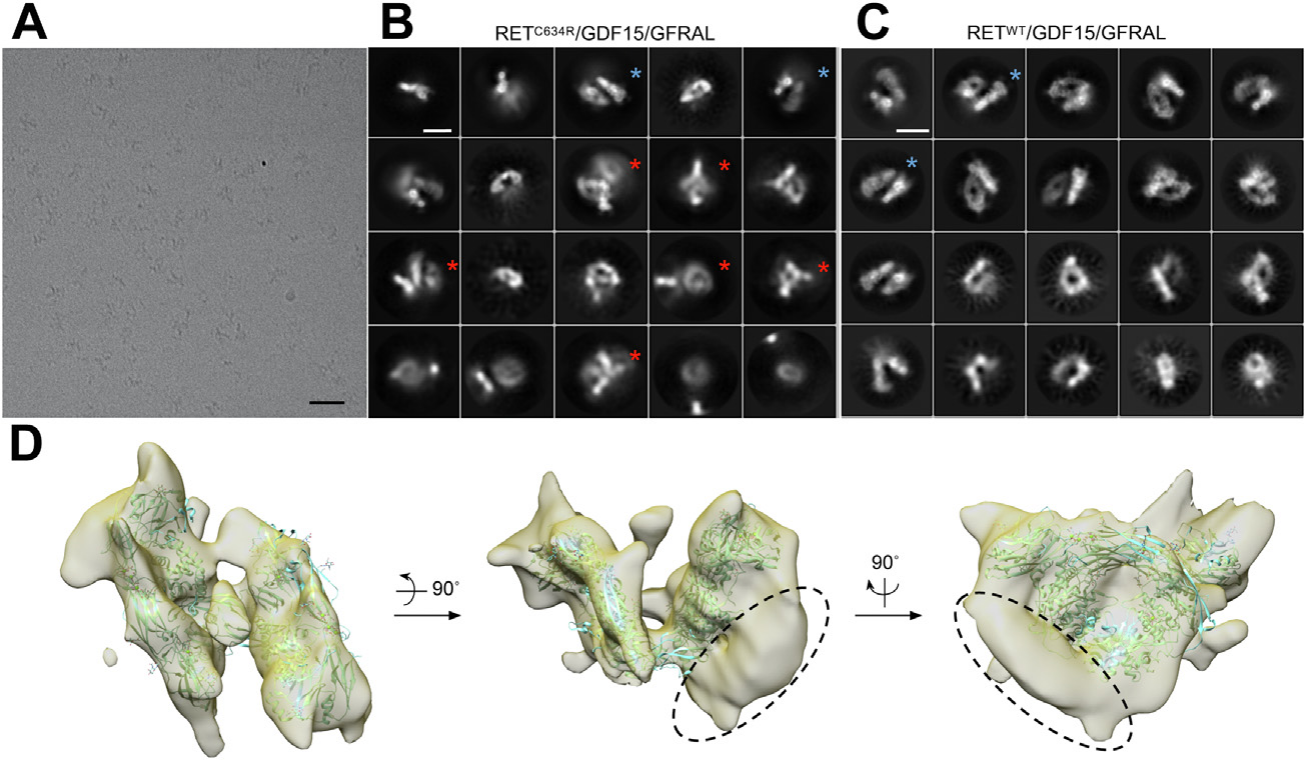
**Cryo-EM analysis of the extracellular domain complex of RET**^**C634R**^**/GDF15/GFRAL.***A*, representative cryo-EM field of RET^C634R^/GDF15/GFRAL. Scale bar represents 100 nm. *B*, 2D class average from 288927 cryo-EM particles of RET^C634R^/GDF15/GFRAL. Scale bar represents 10 nm. *C*, 2D class average from 244,672 cryo-EM particles of RET^WT^/GDF15/GFRAL. Scale bar represents 10 nm. Unique classes of RET^C634R^/GDF15/GFRAL are marked with *red stars* and representative similar classes between RET^C634R^/GDF15/GFRAL and RET^WT^/GDF15/GFRAL are marked with *blue stars*. *D*, different views of a 3D cryo-EM model of RET^C634R^/GDF15/GFRAL. A structure of RET^WT^/GDF15/GFRAL (*cyan*, PDB ID: 6Q2J) is fitted in the density. Extra density observed exclusively for the RET^C634R^/GDF15/GFRAL complex is highlighted with *dotted black circles*. EM, electron microscopy; GFRAL, GDNF receptor α-like.

To obtain higher resolution information regarding the structure of the RET^C634R^/GDF15/GFRAL ECD complex, cryo-EM grids were prepared using the same sample that was used for negative stain EM. The particles in the cryo-EM micrographs exhibit a similar “X” shape to that seen in negative stain EM (Fig. 6*A*). With a set of 288,927 RET^C634R^/GDF15/GFRAL particles, 2D class averages were processed and several classes looked similar to those of the RET^WT^ complex (Fig. 6, *B* and *C*). Additionally, several 2D class averages were observed that are unique to the RET^C634R^ complex (Fig. 6*B*, red stars). These unique 2D classes generally contain a region which is better resolved with the rest of the view blurred, suggesting flexible particles within the classes, so that good alignment was achieved only with part of the particles. 3D classification and refinement with C1 point symmetry result in a 3D model similar to that of a RET^WT^ complex with a final resolution of 10 Å, and the structure of RET^WT^/GDF15/GFRAL (PDB ID: 6Q2J) could be fitted into the density (Fig. 6*D*). Notably, after docking one copy of the RET^WT^/GDF15/GFRAL complex into the map, extra density was observed below one of the two “wings” (Fig. 6*D*, dotted circle), suggesting the presence of additional structurally flexible components to the complex. However, further processing did not help to improve the resolution of the complex by either masked classification and refinement (29), using the NS-EM model as an initial model or by applying D2 symmetry.

### Conformational flexibility of the extracellular domains of RET^C634R^ and the RET^WT^/GDF15/GFRAL complex from MD simulations

**Figure 7:**
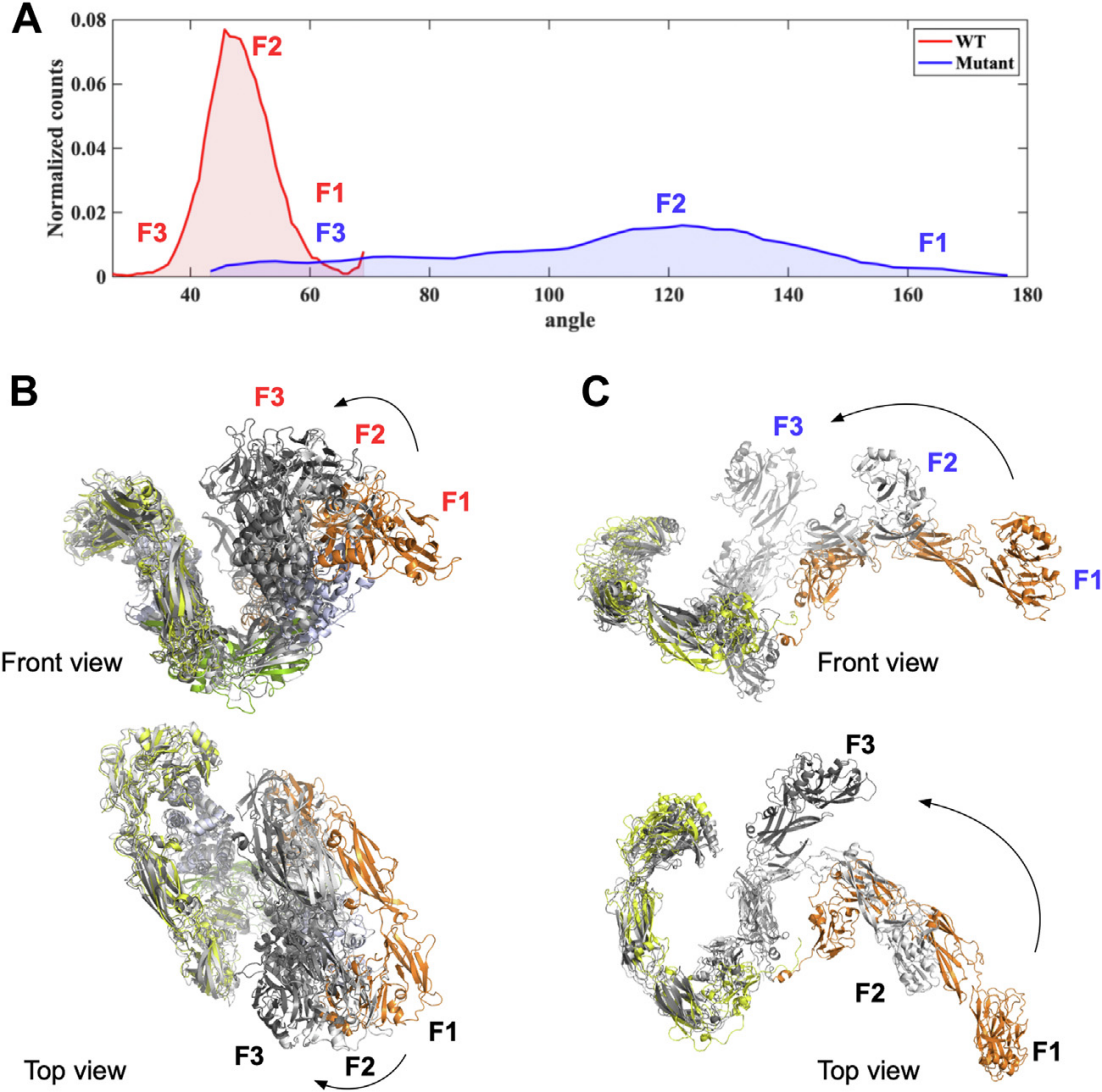
**MD simulation of the extracellular domain complex of RET**^**WT**^**/GDF15/GRFRAL and RET**^**C634R**^**dimer.***A*, distribution of the angles between the two “wings” of the RET^WT^/GDF15/GRFRAL complex (P161^GFRAL^-C77^GDF15^-P161^GFRAL’^, PDB ID: 6Q2J) and the two RET^C634R^ protomers of the RET^C634R^ dimer (V262^RET^-C630^RET^-V262^RET’^). Three representative frames (F1, F2, and F3) of the RET^WT^/GDF15/GRFRAL complex (*B*) and the RET^C634R^ dimer (*C*) aligned along one protomer. GDF15 dimer is colored in *green*, GFRAL in *light blue*, and different RET protomers in *yellow* and *orange*. GFRAL, GDNF receptor α-like.

To further investigate the conformational flexibility of the covalently linked RET^C634R^ dimer and the RET^WT^/GDF15/GFRAL complex, we performed atomistic MD simulations. MD simulations of the RET^WT^/GDF15/GFRAL complex showed that the angle between the two wings oscillates in a normal distribution fashion with the peak in the range of 45 to 50° (Fig. 7). Alignment of the simulation trajectory by backbone revealed that the two RET protomers in the tripartite complex exhibit a higher degree of movement than GDF15 and GFRAL (Fig. S7, and Movie S2), in close agreement with our observations in the cryo-EM maps as described earlier (Fig. 4*C*). MD simulations of the RET^C634R^ mutant dimer predicts a high degree of conformational flexibility, with the angle between the two RET^C634R^ protomers ranging primarily from 110 to 140° (Fig. 7 and Movie S3) but without unraveling of the secondary structure (Fig. S6). Such observation is not surprising as the terminal region of the RET^ECD^ is unstructured and flexible. During the entire simulation runs, the RET^C634R^ dimer did not once adopt a conformation similar to that of RET^WT^ protomers in the RET^WT^/GDF15/GFRAL complex, suggesting that the RET^WT^ conformation is not favored for RET^C634R^ in the absence of its ligands. The further implication of this is that activation may well occur by a different mechanism. It is thus consistent with the formation of the 4:4:4 highly flexible RET^C634R^/GDF15/GFRAL complex described above (Figs. 5 and 6). However, binding of certain ligands, for example GDNF/GFRα1, to RET^C634R^ can apparently force the receptor into a WT-like signaling complex (Fig. 2*A*).

## Discussion

WT RET signaling is regulated by ligand-induced dimerization of the RET ECD, bringing the two intracellular kinase domains close to each other and thus mediating proximity-induced autophosphorylation and activating downstream signaling. The dynamic control of RET complex association and disassociation is essential for normal cellular functions. In MEN2A, RET mutations such as C634R reorganize the disulfide network of the RET ECD such that an intermolecular disulfide bond occurs, enforcing persistent and ligand-independent dimerization of RET. Despite its clinical importance, little is known about the molecular basis for the oncogenic activation of the RET C634R mutant due to a lack of structural studies. In this work, we structurally characterized the extracellular domain of the RET^C634R^ mutant both with and without ligands and showed that the RET^C634R^ dimer can further complex with the different canonical RET-activating ligands in divergent modes that are structurally distinct from RET^WT^ and have the potential to assemble high-order signaling clusters. These discrete structures define the molecular mechanisms through which the RET^C634R^ mutation exerts its oncogenicity.

### The RET^C634R^ ECD dimer adopts an activating conformation in the absence of ligands

**Figure 8:**
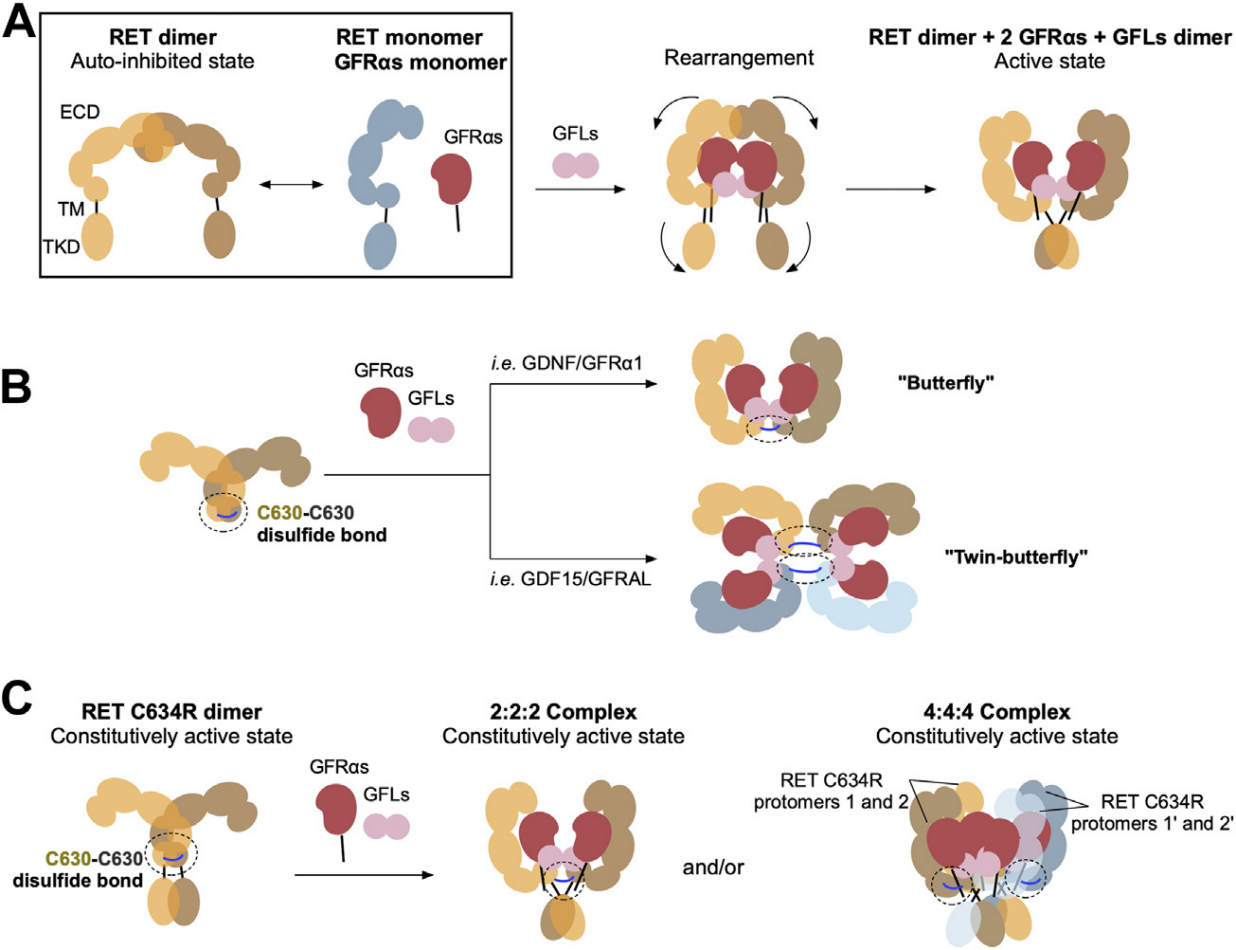
**Proposed models of the dimeric RET**^**C634R**^**and its interaction with its ligands.***A*, cartoon representation of the activation mechanism of full-length RET^WT^ under normal physiology. *B*, two configurations of the reconstituted extracellular domain complexes of RET^C634R^/GDF15/GFRAL and RET^C634R^/GDF15/GFRAL representing ligand-dependent activation mechanisms of RET^C634R^. The two RET monomers are colored in *yellow* and *brown*. Instead of forming a “butterfly” conformation seen in the RET^WT^ complexes, RET^C634R^ complexes with the coreceptors (*red*) and GFLs (*pink*) adopt either a “butterfly” conformation or a novel “twin-butterfly” conformation. *C*, cartoon representation of our proposed model for full-length RET^C634R^ activation by extrapolation from our extracellular domain structures. RET protomer 1 and 1’ (*yellow* and *brown*) forms one RET^C634R^ dimer, while RET protomer 2 and 2’ (*blue* and *light blue*) forms another. GFRAL, GDNF receptor α-like.

In the absence of activating ligands, the extracellular domains of RET^WT^ either exist as inactive monomers or dimerize in an inactive head-to-head conformation (16) (Fig. 8*A*). Our work and other EM studies have shown that activation of RET^WT^ ECD is mediated by the formation of a 2:2:2 “butterfly” complex with its ligands, driving a tail-to-tail conformational rearrangement that promotes proximal kinase domain activation (Fig. 3*B*). We reveal that the aberrant disulfide bond of the oncogenic RET^C634R^ ECD mutant enforces a dimeric “S” conformation that presumably results in constitutive activation of RET signaling in the full-length receptor (Figs. 8*B*, 1, *C* and *E*). The angle between the two docked RET^C634R^ ECD protomers is approximately 140°, which is wider than that of the two RET^WT^ protomers in complex with any of its ligands (PDB ID: 6Q2O, 6Q2N, and 6Q2J) but consistent with the modal value in our MD simulations (Fig. 7, *A* and *C*). Conversely, the distance measured between the P622 residues (ca. 18 Å) is three times shorter (42–50 Å). This implies that the unliganded RET^C634R^ dimer may result in more efficient autoactivation than for a liganded RET^WT^ complex, although the lack of structural information regarding full length RET precludes a comprehensive understanding of how these distances and angles affect the intracellular domains.

Whereas C630 and C634 are located at the end of the flexible C-terminal loop of RET^ECD^, the other cysteine residues, such as C609, C611, C618, and C620 whose mutations are also associated with MEN2A and are cancer-inducing, albeit with better prognosis, sit in two strands of an antiparallel β-sheet. In WT RET, C609-C620 and C611-C618 form two intramolecular disulfide bonds. Interestingly, we found that expression of RET^C620R^ ECD using the same approach and conditions as for RET^C634R^ ECD also yielded a disulfide-crosslinked dimer after Fc-tag removal (Fig. S8). However, the extent of dimerization was less than for RET^C634R^, which correlates with the reduced oncogenic potency of RET^C620R^ (3). We hypothesize that the formation of the C609-C609 intermolecular disulfide bond in RET^C620R^ is less favored due to structural constraints, which may be the same for other cysteine mutation-induced oncogenic dimers of RET and explain why C634 mutations are the most aggressive. Equally, some of these dimerized RET mutants may not pass through the protein production machinery as efficiently, in agreement with previous reports that the C609, C611, C618, and C620 mutations impair RET folding and maturation (30, 31, 32).

### Divergent activation of RET^C634R^ is mediated by ligand-dependent complexation

We show that RET^WT^ ECD readily forms 2:2:2 tripartite complexes with both GDF15/GFRAL and GDNF/GFRα1 (Figs. 2 and 3), in accordance with previous reports (16, 33). We observed multiple conformations of the RET^WT^/GDF15/GFRAL complex using cryo-EM, revealing that the “wings” undergo a concerted twisting and bending motion (Fig. 4). Our MD simulations corroborate this finding and predict that the degree of these movements would be even larger at biologically relevant temperatures (Fig. 7). In particular, we found that, within the tripartite complex, the RET^ECD^ undergoes the greatest degree of movement (Fig. 4*C*), which is consistent with findings from previous studies of the RET^WT^/NTRN/GFRα2 (27) and the zebrafish RET^WT^/GDNF/GFRα1 (34) complexes. Such conformational flexibility might be anticipated for a protein capable of binding as diverse range of ligands as RET but may also have limited the resolution of the cryo-EM map reconstructed with the data used in this study.

**Figure 9:**
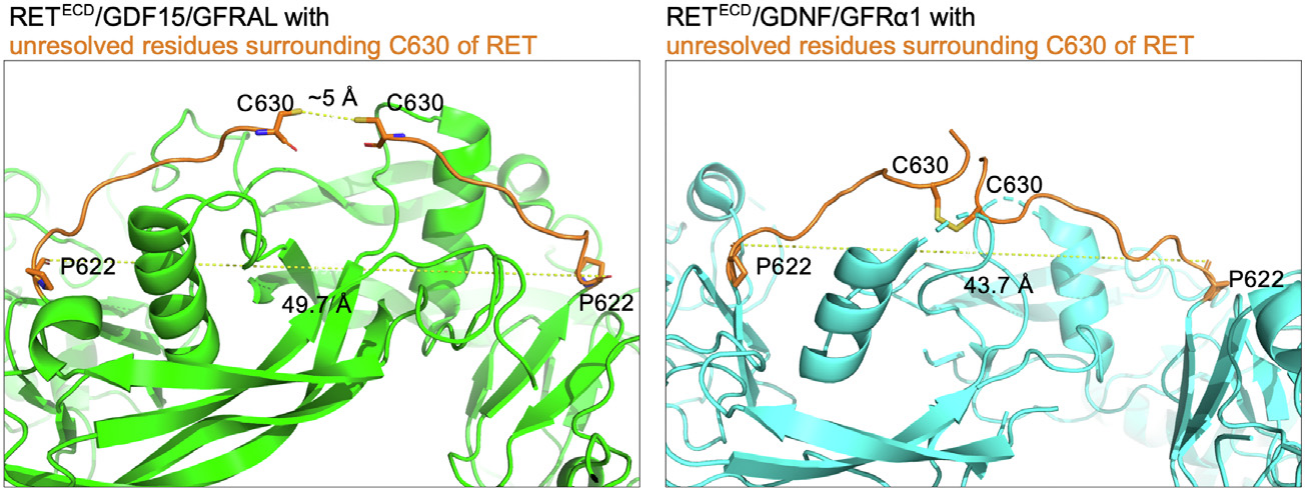
**Analysis of the potential C630-C630 disulfide bond formation for the RET**^**C634R**^**/GDF15/GFRAL and RET**^**C634R**^**/GDNF/GFRα1 extracellular domain complexes in a 2:2:2 configuration.** A polypeptide chain of 8 and 10 unresolved amino acids is added to the C-terminal tail of each RET^ECD^ protomer in RET^WT^/GDF15/GFRAL (PDB code 6Q2J) and RET^WT^/GDNF/GFRα1 (PDB code 6Q2N) using Coot, respectively. Structural comparison and distance measurement were performed using PyMOL. ECD, extracellular domain; GFRAL, GDNF receptor α-like.

We found that mutant RET^C634R^ ECD is capable of forming both the archetypal 2:2:2 tripartite RET-ligand complex but also higher order complexes that have not been previously reported. We have assigned the major higher order complex as a 4:4:4 tripartite complex with a back-to-back twin butterfly configuration. Our studies suggest that, in contrast to RET^WT^, the preference for RET^C634R^ to adopt these distinct complex configurations is ligand dependent. In the published structures of the RET^WT^/GDF15/GFRAL (PDB ID: 6Q2J) and RET^WT^/GDNF/GFRα1 (PDB ID: 6Q2N) complexes, the distance between the last resolved residues (P622) of each RET^ECD^ protomer are ∼50 Å and ∼44 Å, respectively. In the case of RET^C634R^/GDNF/GFRα1, we were able to model in the unresolved C-terminal residues of RET^ECD^ (622–632) as disordered chains containing the mutant C630-C630 disulfide bond. However, this is not at all possible for RET^C634R^/GDF15/GFRAL, on account of the increased RET^ECD^-protomer separation, explaining the ligand-dependent architecture of the RET^C634R^ complexes that we observed (Fig. 9). Furthermore, the RET^C634R^/GDNF/GFRα1 complex is more thermally stable relative to RET^WT^/GDNF/GFRα1 and this is likely explained by the entropic benefit provided by the C630-C630 RET stapling (Fig. 2*B*). If these distinct arrangements of the RET^C634R^ ECD complexes translate to its full-length form *in vivo*, they could impact on the extent of its activation in different tissues. We anticipate that the complexes characterized here are specific to the C634R mutation and that the other oncogenic cysteine mutations of RET may impact on the ligand binding in different ways.

### Relevance to cancer

Antibody-based studies have identified that GFRAL is expressed in multiple cancer tissues (Human Protein Atlas available from http://www.proteinatlas.org/) (35) and its signaling *via* RET^C634R^ may be biologically relevant under disease conditions. The ECD of RET^C634R^ clearly forms a twin-butterfly complex with GDF15/GFRAL with 4:4:4 stoichiometry (Figs. 2 and 5), and we speculate that about 30% of the RET^C634R^/GDNF/GFRα1 also forms a 4:4:4 complex. The structures seen in this study obviously lack the spatial constraints imposed by the cell membrane. In our low-resolution NS-EM model, the CRDs of the docked dual-hexameric complex sit directly opposite from each other (Fig. 5*E*). However, given how flexible the RET^C634R^ cross-linked dimer is (Fig. 7), the hexameric complexes in the membrane-bound full length 4:4:4 complex might be able to bend toward each other to allow more efficient anchoring of the TM domains into the membrane (Fig. 8*C*). Such a collapsed “twin-butterfly” 4:4:4 complex might even lead to the formation of locally clustered higher-order oligomers (16, 36) that have been characterized for related receptors such as of Eph receptors (37) and endothelial growth factor receptor (38).We speculate that the change of conformation from “butterfly” to “twin-butterfly” leads to different signaling outcomes and intensities (39) and thus provides a structural explanation for oncogenesis. However, as we have only observed these configurations in the context of the isolated extracellular domains, it remains to be seen whether such complexes do form *in vivo* and how they might influence signaling in cancer. Nonetheless, these insights pave the way for further investigations into RET gain-of-function activation under disease conditions and potentially for the discovery of cancer therapies targeting the extracellular domain of RET^C634R^ as well as the other dimerization-inducing oncogenic cysteine mutations.

## Experimental procedures

### DNA constructs, protein expression, and purification

The gene encoding human RET^ECD^ (residues 1–635) bearing the C634R, C86R, and C216S mutations (RET^C634R^) with a C-terminal Thrombin protease cleavage site, Fc, and His_8_-Flag tags (RET^C634R^-Fc) was cloned into pcDNA3.1 vector in frame with the cytomegalovirus promotor (Tables S1 and S2). WT RET^ECD^ (residues 1–635) was subcloned into pcDNA3.1 vector with a Tobacco Etch Virus protease cleavage site and His_8_-Flag tags (RET^WT^). Human GDF15 (residues 195–308) was cloned into the same vector with a modified N-terminal Fc tag, followed by a thrombin cleavage site, and this construct was used for cotransfection with a separate construct for the expression of modified Fc as described previously (25, 40). The construct generation and expression procedures of RET^WT^, Fc-GDF15, and human GFRAL (residues 19–351) with C-terminal His_8_-twin Strep tags were the same as previously described (25). Human GDNF (residues 142–275) and human GFRα1 (residues 25–429) were subcloned into pk503.9 vector with N-terminal Flag-His_8_ tags followed by a thrombin cleavage site. The over-expression of recombinant RET^C634R^-Fc, RET^WT^, and Fc-GDF15 were achieved using transient expression in adherent HEK293T cells (American Type Culture Collection, CRL-3216), while GFRAL, GDNF, and GFRα1 were expressed using the baculovirus-infected insect cell system in *Trichoplusia ni* High Five (Hi5) and *Spodoptera frugiperda* (*Sf* 9) insect cells (Thermo Fisher Scientific) maintained as suspension cultures. All proteins were secreted into the cell culture medium and the culturing medium containing the secreted proteins was harvested 7- and 5-days post-transfection for RET^C634R^-Fc or RET^WT^ and Fc-GDF15, respectively and after 3 days for GFRAL, GDNF, and GFRα1. The reconstruction of the GDNF/GFRα1 complex was achieved either by coexpression and purification or the assembly of the separately purified proteins.

To purify RET^C634R^, Protein A resin (GenScript) was used to immobilize RET^C634R^-Fc for 2 hours, followed by a thorough washing step using washing buffer containing 20 mM Hepes pH 7.5, 150 mM NaCl, and 1 mM CaCl_2_. Biotinylated thrombin (Merck Millipore) was added to the resin and the sample was incubated at 4 °C for 12 h without agitation. After protease cleavage, the flow-through containing the cleaved proteins was collected and incubated with pre-equilibrated Strep-Tactin resin (IBA Lifesciences) to remove any unbound thrombin for 30 min at room temperature (RT). The supernatant was then collected and concentrated using Amicon concentrators (50 kDa). A final polishing step was performed by size-exclusion chromatography (SEC) on a Superdex 200 increase 10/300 Gl column (GE Healthcare) in 20 mM Hepes pH 7.5, 100 mM NaCl, and 1 mM CaCl_2_. Each elution fraction was subject to nonreducing SDS PAGE for purity evaluation and the peak fraction was directly used to prepare EM grids. For storage, fractions containing the dimeric RET^C634R^ were combined and concentrated with Amicon concentrators (50 kDa). The final concentrated samples were stored at −70 °C in SEC buffer containing 7.5% glycerol.

The purification of RET^WT^, Fc-GDF15, and GFRAL was performed as described before (25). In brief, RET^WT^ was purified using a two-step purification protocol, which included Ni-NTA (Qiagen) and anti-Flag (GenScript) affinity purifications, and the final sample was eluted from anti-Flag resin using poly-Flag peptide (Bimake). The peptide was removed by buffer exchange using micro bio-spin columns (Bio-Rad). To purify Fc-GDF15, medium containing the secreted protein was incubated with Protein A resin and eluted using low-pH buffer containing 100 mM sodium citrate pH 3.1 and 100 mM NaCl, followed by immediate neutralization to pH 7.5 using 1 M Tris pH 8.8. For storage, purified Fc-GDF15 was buffer-exchanged into Tris-buffered saline containing 7.5% glycerol. Strep-Tactin resin was used to affinity purify GFRAL and the bound protein was eluted using 5 mM d-desthiobiotin. The eluate was further purified by SEC on a Hiload Superdex 200 pg 16/600 (GE Healthcare) in 20 mM Hepes pH 7.2, 150 mM NaCl and buffer exchanged into storage buffer containing 20 mM Hepes pH 7.2, 150 mM NaCl, and 7.5% glycerol. To purify coexpressed GDNF/GFRα1 or GDNF, Ni-NTA resin was used to capture His-tagged proteins and the bound protein was eluted with TBS containing 300 mM imidazole. The eluate was incubated with thrombin protease for tag cleavage overnight at RT and was then concentrated using Amicon concentrators (10 kDa or 5 kDa) and purified on a Superdex 200 increase 10/300 Gl column (GE Healthcare) in 20 mM Hepes pH 7.5, 100 mM NaCl. The peak fractions containing the desired proteins were combined, concentrated, and were stored at −70 °C in SEC buffer containing 7.5% glycerol. GFRα1 was purified similarly to that of the GDNF/GFRα1 complex but without protease cleavage.

### Bio-layer interferometry technology system

A bio-layer interferometry technology system instrument (FortéBio) was used to measure the binding affinity between RET^C634R^ and Fc-GDF15/GFRAL, and the experimental set up was as previously described (25). Fc-GDF15 (15 μM) was first immobilized on anti-hIgG Fc capture biosensors (FortéBio) and the biosensors bound with Fc-GDF15 were then saturated by binding to GFRAL (3 μM). Various concentrations of RET^C634R^ (0.3, 1.2, 2.8, 7, 14, 28, and 42 μM) were used for the second binding step and the saturation signals at the end of the second binding steps were analyzed as a function of RET^C634R^ concentration on a logarithmic scale. The dissociation constant (*K*_d_) was obtained through fitting a nonlinear regression sigmoidal model using GraphPad Prism 8.

### BN PAGE

The RET/Fc-GDF15/GFRAL tripartite complexes were prepared by incubating Fc-GDF15 dimer (2 μM) with GFRAL (4 μM) for 15 min, followed by the addition of either RET^WT^ monomer (4 μM) or RET^C634R^ dimer (2 μM) to a final volume of 10 μl. Similarly, the RET/GDF15/GFRAL complex was prepared by adding 0.01 U thrombin protease to the GDF15/GFRAL complex prior to the addition of RET. The formation of the RET/GDNF/GFRα1 complex was performed by incubating RET and copurified GDNF/GFRα1 at a molar ratio of 1:1 at the same concentrations. The mixtures were kept at RT for 30 min to allow complex formation. BN PAGE loading buffer was added to each sample before electrophoresis, which was performed at 100 V and 4 °C for 3.5 h (25, 41, 42). Gels were destained with 20% methanol and 10% acetic acid.

For the temperature-dependent stability measurement, the RET/GDF15/GFRAL and RET/GDNF/GFRα1 complexes were reconstituted as described above, aliquoted into PCR tubes, and heated for 5 min at 50 °C, 54 °C, 57 °C, 59 °C, 60 °C, 64 °C, 70 °C and 54 °C, 60 °C, 64 °C, 67 °C, 70 °C, 72 °C, 76 °C, 80 °C, 85 °C, respectively. After incubation, the samples were immediately placed on ice for 10 min, centrifuged, and mixed with BN PAGE loading buffer. The electrophoresis was performed as described above. The band intensities of the complexes were measured using ImageJ (43). Normalized intensities were analyzed against the different temperature points, and the melting temperature (*T*_m_) was obtained through fitting a nonlinear regression sigmoidal model using GraphPad Prism 8.

### Complex reconstitution and purification for structural studies

To form the RET^WT^/Fc-GDF15/GFRAL complex, Fc-GDF15 and GFRAL were first incubated at a molar ratio of 1:2 for 15 min at RT. RET^WT^ was then added to the mixture so that the final molar ratio of RET^WT^, Fc-GDF15 dimer, and GFRAL was 2:1:2. The sample was purified using SEC after 30 min postincubation on a Superose 6 increase 10/300 Gl column in 20 mM Hepes pH 7.5, 100 mM NaCl, and 1 mM CaCl_2_. For the preparation of the RET^C634R^/GDF15/GFRAL complex, Fc-GDF15 and GFRAL at a molar ratio of 1:2 were first incubated together with 0.055 U thrombin per 100 μg Fc-GDF15 for 15 min at RT before the addition of the RET^C634R^ dimer. The final molar ratio of RET^C634R^ dimer, GDF15 dimer, and GFRAL was 1:1:2. The protein mixture was kept at RT for an additional 30 min and the complex separated from thrombin using SEC on a Superdex 200 increase 10/300 Gl column in 20 mM Hepes pH 7.5, 100 mM NaCl, and 1 mM CaCl_2_. For both complexes, the purity of each fraction from SEC was assessed using BN PAGE and the peak fractions were directly used to prepare electron microscopy grids without concentrating. For protein storage or other applications, fractions containing the complexes were pooled and concentrated with Amicon concentrators (50 kDa).

### Size exclusion chromatography-coupled multiangle static laser light scattering

SEC-MALS was used to characterize the particle distribution and the oligomeric state of RET^C634R^ dimer, RET^C634R^/GDF15/GFRAL, and RET^WT^/GDF15/GFRAL complexes. All measurements were performed with a HPLC system (Shimadzu) on a Superdex 200 10/300 Gl column with a flow rate at 0.25 ml/min at RT in SEC buffer containing 20 mM Hepes pH 7.5, 100 mM NaCl, and 1 mM CaCl_2_. The MALS system was equipped with MiniDAWN TREOS light scattering and Optilab rEX refractive index detectors (Wyatt Technology Corp) The data was first analyzed using ASTRA 6 software (Wyatt Technology Corp) and was exported and replotted using GraphPad Prism 8.

### NS-EM sample preparation

Negative stain grids were glow-discharged using either a Pelco Easiglow unit with 10 mA for 30 s or a Quorum Emitech K100X Glow discharge unit with 30 mA for 45 s. For negative stain EM experiments, 3 μl of protein samples at 0.01 mg/ml for RET complexes or 0.005 mg/ml for RET^C634R^ dimer were first applied to the glow-discharged 300 mesh copper grids with carbon coating for 1 min. The grids were blotted, washed once quickly with a 10 μl drop of 2% (w/v) uranyl acetate (UA) solution, blotted again, and incubated with a second 10 μl drop of UA for 45 s, followed by a final blotting step to remove the stain. The grids were left to air dry for 5 min at RT before imaging or storage.

### NS-EM data processing

For the RET^C634R^ dimer, 40 micrographs were collected using a Tecnai F20 TEM (FEI, 200 kV) at a nominal magnification of 50,000 magnification resulting in 2.25 Å/pixel. Grids of RET^C634R^/GDF15/GFRAL were imaged using the same microscope and the same settings with a total of 68 micrographs collected. NS-EM data processing was performed using EMAN2.3 (44). After micrograph evaluation, contrast transfer function (CTF) estimation and structure factor calculation were carried out. A total of 3490 and 11,278 particles were manually picked for the RET^C634R^ dimer and the RET^C634R^/GDF15/GFRAL complex, respectively. Reference-free 2D classification, initial model building, and single-map refinement were performed in EMAN2.3. The initial model building was performed with C2 symmetry for the RET^C634R^ dimer and the same symmetry was applied in subsequent refinement steps for the RET^C634R^ dimer. For the 3D model reconstruction of the RET^C634R^/GDF15/GFRAL complex, no symmetry was applied in the initial model building and the model refinement.

### Cryo-EM sample preparation

Cryo-EM grids were glow discharged using the same method as for the NS-EM grids. To prepare the specimens for data collection, 3 μl of protein samples at 0.12 mg/ml for both RET^WT^/GDF15/GFRAL and RET^C634R^/GDF15/GFRAL were applied to glow-discharged Quantifoil R1.2/1.3300 mesh gold grids for 5 s before blotting. The grids were prepared using either a Vitrobot Mark IV with 6 s blot time (blot force 6) or a Leica EM GP with 1.5 s blot time at 4 °C with 80% humidity.

### RET^WT^/Fc-GDF15/GFRAL cryo-EM data processing

For RET^WT^/Fc-GDF15/GFRAL, the micrographs were collected on a Titan Krios electron microscope (300 kV) with a Falcon 3EC direct electron detector in integrative mode at 1.065 Å/pixel at 75,000 nominal magnification. A total of 6563 movies were collected with a defocus range of −1.6 to −3 μm with a dose per fraction of 1.5 e/Å^2^ and a total dose of 60.3 e/Å^2^ (Table S3). Most steps of image processing were performed using RELION 3 (Fig. S9). MotionCorr2 was used to perform beam-induced motion correction of the micrographs (45) and GCTF was used for the CTF estimation (46). The corrected micrographs were subject to particle auto-picking using crYOLO with the general model (47). A total of 1,135,874 particles were extracted after rescaling to a pixel size of 2.13 Å/pixel. Two rounds of 2D and 3D classification were performed to separate particles with different qualities. Two processing routes were performed for the 3D model reconstruction. In one route, 21 images from 2D class averages with 302,004 particles were selected for initial model building and one of the five initial models was used as a reference map for 3D classification. In the other route, a 30 Å map was generated based on the published cryo-EM structure of RET^WT^/GDF15/GFRAL (PDB ID: 6Q2J) using Chimera (48) which was then 60 Å low-pass filtered and used as a reference map for subsequent 3D classification and refinement with C2 symmetry. The structure of RET^WT^/GDF15/GFRAL (PDB ID: 6Q2J) was fitted into the final density map using Chimera. Additional glycans (N-acetylglucosamine) at different glycosylation sites, not present in the published structure (PDB ID: 6Q2J), were added using Coot (49) through manual model building to fit in the densities of the glycans in the refined density map. Analysis of different 3D classes was performed in Chimera.

### Rigid-body flexible fitting into cryo-EM density maps

To prepare the structure of the RET^ECD^/GDF15/GFRAL^ECD^ complex (PDB ID: 6Q2J), glycans and ions were first removed from the original structure. Rigid bodies were identified using Ribfind (50). The individual domains of RET^ECD^, one GFRAL^ECD^ monomer with one GDF15 monomer were set as rigid bodies with a Ribfind cut off of 30% for flexible fitting into individual cryo-EM maps at a resolution of 11 Å using Flex-EM (51). The final models were refined using PHENIX real-space refinement (52) and assessed by rigid fitting into the corresponding cryo-EM maps in Chimera. The structures were superimposed onto the GDF15 dimers and morphing was performed to evaluate the conformational change.

### RET^C634R^/GDF15/GFRAL cryo-EM data processing

For RET^C634R^/GDF15/GFRAL, the micrographs were collected on a Titan Krios electron microscope (300 kV) with a BioQuantum967 (K2 summit camera, Gatan) in counting mode at 1.07 Å/pixel at 130,000 nominal magnification. A total of 2852 movies were collected with a defocus range of -1.6 to -3 μm with a dose per fraction of 1.26 e/Å^2^ and a total dose of 62.88 e/Å^2^ (Table S3). Following motion correction and CTF estimation as described earlier using RELION 3, 309,003 particles were autopicked using crYOLO, rescaled, and extracted with 2.675 Å pixel size. The bad particles were removed through four rounds of 2D class averaging, and 18 images from 2D classes with 281,224 particles were selected for 3D classification (C1 point symmetry) using the low-pass–filtered map of RET^WT^/GDF15/GFRAL (PDB ID: 6Q2J) as described earlier as a reference map. One of the three maps from 3D classification was selected as the reference map for auto-refinement (C1 point symmetry).

### MD simulation

We performed classical atomistic MD simulations on the WT RET/GDF15/GFRAL complex (PDB ID: 6Q2J) and the RET^C634R^ dimer. The cross-linked RET^C634R^ dimer was constructed by docking two copies of RET monomers adapted from the RET/GDF15/GFRAL complex (PDB ID: 6Q2J) in the reconstructed NS-EM map of the RET^C634R^ dimer. Thirteen additional amino acids (^623^EDIQDPLCDELRR^635^) were added to the C-terminal region of RET^ECD^ using Coot, which are absent in the PDB file used, with a disulfide bond between the C630 residues of the two RET^C634R^ protomers. Web-based CHARMM-GUI tools were used to construct the model systems (53, 54, 55). Each model system consisted of protein, structurally resolved calcium ions and paucimannose modeled at the known glycosylation sites (56, 57, 58) and was solvated in TIP3P water molecules, containing sodium and chloride ions at a concentration of 100 mM. The model system sizes ranged from ∼550,000 to ∼1,000,000 atoms depending on the simulation box sizes. Due to the irregular shape of the protein, the simulation box was rectangular and protein rotation was prevented by keeping harmonic constraints on individual Cα-atoms of residues: 432, 496, and 506 from RET protomers. The strength of the constraints varied from 2000 kJ mol^-1^ nm^-2^ to 50,000 kJ mol^−1^ nm^−2^ to ensure the constraints do not affect the results. Multiple independent 100 to 400 ns MD simulations of WT (∼1.18 μs) and mutant (∼1.49 μs) model systems were performed using simulation software GROMACS 2020.2 (59, 60, 61) and CHARMM36 (62, 63) forcefield. After minimization and equilibration of the systems, the pressure and temperature were kept stable using Nose-Hoover thermostat (64, 65) and Parrinello-Rahman barostat (66, 67). The timestep of simulations was 2 fs, which was achieved with LINCS algorithm (68), and the long-range electrostatics was handled with Particle-mesh-Ewald (69) as implemented in GROMACS. The simulation trajectories were analyzed using software VMD (70).

## Data availability

All representative data are contained within the article.

## Supporting information

This article contains supporting information.

## Conflict of interest

Authors declare that they have no competing interests.
