# Supplementary material for: Unexpected structures formed by the kinase RET C634R mutant extracellular domain suggest potential oncogenic mechanisms in MEN2A
Source: J Biol Chem. 2022 Aug 17;298(10):102380. doi: 10.1016/j.jbc.2022.102380 (PMC9490035; doi:10.1016/j.jbc.2022.102380)
Supplement: Supplemental information [file mmc4.docx]

Supplementary Information

**Expression and analysis of RET^C620R^-Fc and un-tagged RET^C620R^**

HumanRET^ECD^-Fc harbouring the C620R, C86R and C216S mutations were sub-cloned into pcDNA3.1 vector with a Thrombin protease site and C-terminal His_8_-Flag tags. For the expression test, HEK293T cells were seeded in 6-well cell culture plates in Dulbecco's Modified Eagle Medium (DMEM, Sigma-Aldrich, D6429) containing 10% fetal bovine serum (FBS, Gibco) 24-hr prior to the transfection. For the transient transfection, the conditioned medium was replaced with fresh DMEM with 4% FBS. Transfection cocktails were prepared by mixing 4.3 μg DNA with 13 μg polyethylenimine (PEI) (Polysciences Europe GmbH) in 500 μl DMEM medium without FBS per well and incubated at RT for 8 min before adding to the cells. Protein expression was allowed for 7 days at 37 ˚C, 5% CO_2_. Supernatant containing the secreted proteins was collected and incubated with 30 μl pre-equilibrated Protein A resin for 2 h in the cold room. The beads were washed with washing buffer containing 20 mM HEPES pH 8, 150 mM NaCl, 1 mM CaCl_2_ and 0.05% Tween-20. Thrombin protease (1 U) was added to the samples and incubated overnight to release the bound proteins. The next day, supernatant containing the cleaved proteins was collected and analyzed using SDS PAGE and western blotting under reducing and non-reducing conditions with anti-RET(C3)-HRP antibody (Santa Cruz Biotechnology).

Figure S1. Temperature-dependent stability measurement of RET^C634R^/GDNF/GFRα1 (A, upper panel), RET^WT^/GDNF/GFRα1 (A, lower panel), RET^C634R^/GDF15/GFRAL (B, upper panel) and RET^WT^/GDF15/GFRAL (B, lower panel).

Figure S2. Characterization of the RET^WT^/GDF15/GFRAL complexes using SEC MALS. The calculated average molecular weight is 393 kDa. BN PAGE analysis of the elution fractions of RET^WT^/GDF15/GFRAL are shown in lower panel, suggesting that the second peak in upper panel corresponds primarily to RET^WT^/GDF15/GFRAL.

Figure S3. Semilog plot of binding of RET^C634R^ for the Fc-GDF15/GFRAL complex. Saturation binding curve fitted with nonlinear regression sigmoidal model. The concentration of RET^C634R^ is plotted on a logarithmic scale. CI: Confidence interval.

Figure S4. Cryo-EM density map of RET^WT^/GDF15/GFRAL with close-up views at glycosylation sites. The structure of RET^WT^/GDF15/GFRAL (grey, PDB ID: 6Q2J) is fitted in the density. One N-Acetylglucosamine (GlcNAc) is placed at each glycosylation sites to fit the density using Coot.

Figure S5. Views of a 3D initial model of the RET^C634R^/GDF15/GFRAL complex reconstructed from negative stain EM data.


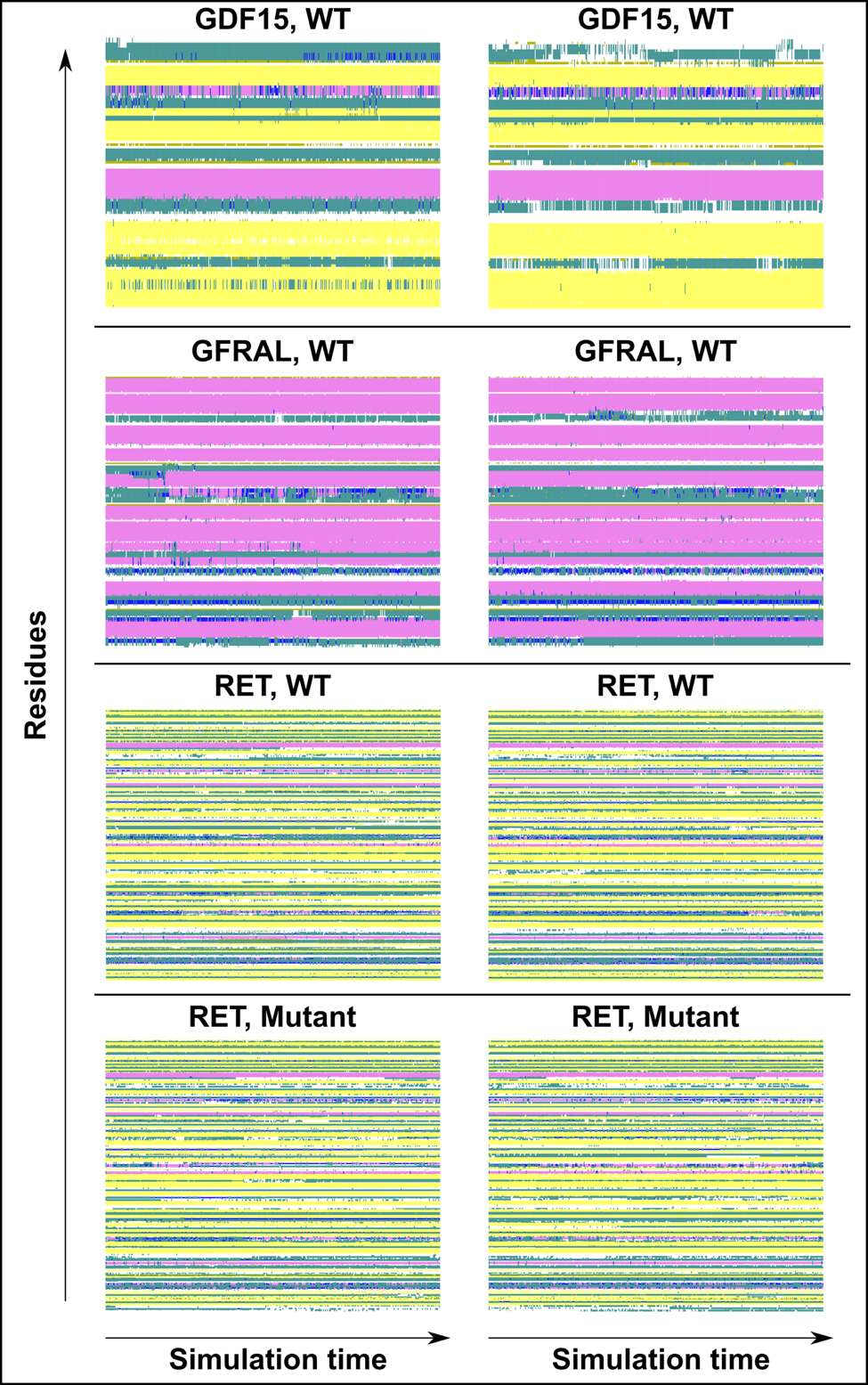


Figure S6. Stability of the protein secondary structure in simulations of the RET^WT^/GDF15/GFRAL complex and RET^C634R^ dimer. Data is shown from longest simulations (440 ns in RET^WT^/GDF15/GFRAL and 330 ns in RET^C634R^ dimer simulations). Colors represent different types of secondary structures: teal (turn), yellow (extended configuration or β-sheet), golden (isolated bridge), lilac (α-helix), blue (3_10_ helix) and white (coil). Results from the two protomers of each protein component in the complex are shown separately in the left and right panels.


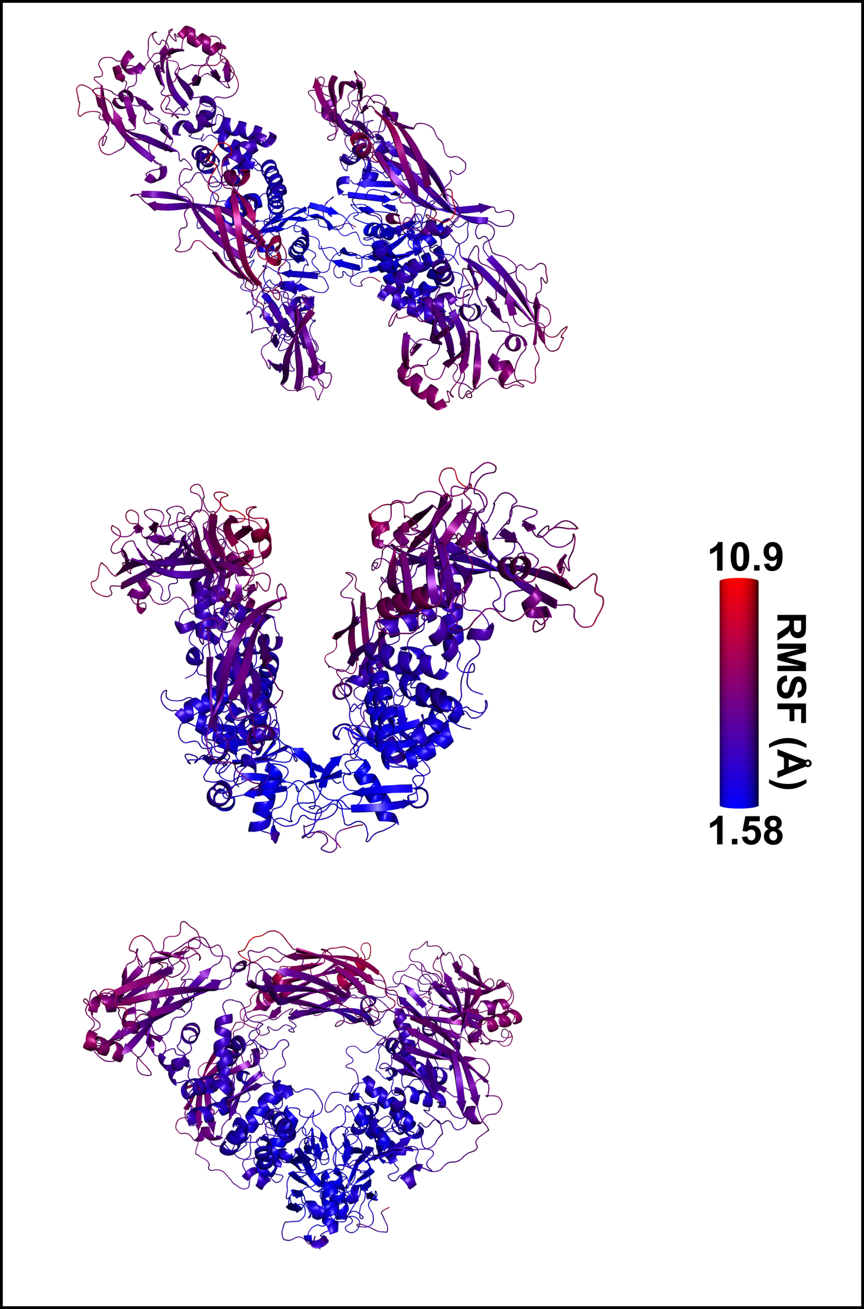


Figure S7. Root mean square fluctuation (RMSF) of RET^WT^/GDF15/GRFRAL combining all simulation runs (1.18 μs) showing top, front and side views.

Figure S8. Anti-RET WB showing RET^C634R^-Fc and RET^C620R^-Fc before and after Fc tag removal. The Thrombin protease was added for the Fc tag removal and the RET^C634R^ dimer is marked with red stars. Samples were prepared under non-reducing (- DTT) and reducing conditions (+ DTT). B: Protein A beads; S: Supernatant sample without beads.

Figure S9. Flow chart of the image-processing pipeline for the cryo-EM data of hRET^ECD^/hGDF15/hGFRAL^ECD^. A total of 6563 dose-fractionated movies were collected on a 300-keV Titan Krios with a Falcon III camera. Alignment and CTF estimation and correction were performed using RELION3. A representative micrograph is shown after motion correction. Particles were autopicked using crYOLO and bad particles were removed after 2D classification. Twenty highest-populated 2D class averages are shown (image also shown in Fig. 6C). Scale bar = 10 nm. Route 1 (right panel): with a set of 302004 particles, initial models were built using RELION-3. One of the initial maps was low-pass-filtered (60 Å) and was used as a reference map for subsequent 3D classification and one subset with 142083 particles were select for auto-refinement. Route 2 (left panel): a 30 Å map was built based on the cryo-EM structure of hRET^ECD^/hGDF15/hGFRAL^ECD^ (PDB code 6Q2J) using Chimera. The map was low-pass filtered and used as a reference map for 3D classification and auto-refinement, with a dataset of 244672 particles. All data processing was performed using RELION-3 unless otherwise indicated.

Table S1. List of primers and methods used for RET^ECD^ mutagenesis.

|  | **Primer (forward)** | **Primer (reverse)** | **Cloning method** |
| --- | --- | --- | --- |
| RET(C87R) | CAACTGGATCCGCATCCAGGAG | TTCTCATGCAGCCGTGTG | Q5 (NEB) Site-Directed Mutagenesis |
| RET(C216S) | CCCTTCCGCTCCGCCCCGGAC | CAGACCCTCACCCTCCAGGAGC | Q5 (NEB) Site-Directed Mutagenesis |
| RET(C620R) | GTGCTTCAGGGAGCCCGAAGACATCCAGG | GGCTCCCTGAAGCACTTCTCCTCCTCAGGG | In-Fusion cloning (Takara Bio) |
| RET(C634R) | CGAGCTGAGGCGCGAGTTCCTGGTTCCG | TCGCGCCTCAGCTCGTCGCACAGTGG | In-Fusion cloning (Takara Bio) |

Table S2. Sequence of the RET^ECD^ C634R-Fc construct used in this study.

| Construct | Sequence |
| --- | --- |
| RET^C634R^-thrombin site-Fc-HF | atggcgaaggcgacgtccggtgccgcggggctgcgtctgctgttgctgctgctgctgccgctgctaggcaaagtggcattgggcctctacttctcgagggatgcttactgggagaagctgtatgtggaccaggcagccggcacgcccttgctgtacgtccatgccctgcgggacgcccctgaggaggtgcccagcttccgcctgggccagcatctctacggcacgtaccgcacacggctgcatgagaacaactggatccgcatccaggaggacaccggcctcctctaccttaaccggagcctggaccatagctcctgggagaagctcagtgtccgcaaccgcggctttcccctgctcaccgtctacctcaaggtcttcctgtcacccacatcccttcgtgagggcgagtgccagtggccaggctgtgcccgcgtatacttctccttcttcaacacctcctttccagcctgcagctccctcaagccccgggagctctgcttcccagagacaaggccctccttccgcattcgggagaaccgacccccaggcaccttccaccagttccgcctgctgcctgtgcagttcttgtgccccaacatcagcgtggcctacaggctcctggagggtgagggtctgcccttccgctccgccccggacagcctggaggtgagcacgcgctgggccctggaccgcgagcagcgggagaagtacgagctggtggccgtgtgcaccgtgcacgccggcgcgcgcgaggaggtggtgatggtgcccttcccggtgaccgtgtacgacgaggacgactcggcgcccaccttccccgcgggcgtcgacaccgccagcgccgtggtggagttcaagcggaaggaggacaccgtggtggccacgctgcgtgtcttcgatgcagacgtggtacctgcatcaggggagctggtgaggcggtacacaagcacgctgctccccggggacacctgggcccagcagaccttccgggtggaacactggcccaacgagacctcggtccaggccaacggcagcttcgtgcgggcgaccgtacatgactataggctggttctcaaccggaacctctccatctcggagaaccgcaccatgcagctggcggtgctggtcaatgactcagacttccagggcccaggagcgggcgtcctcttgctccacttcaacgtgtcggtgctgccggtcagcctgcacctgcccagtacctactccctctccgtgagcaggagggctcgccgatttgcccagatcgggaaagtctgtgtggaaaactgccaggcattcagtggcatcaacgtccagtacaagctgcattcctctggtgccaactgcagcacgctaggggtggtcacctcagccgaggacacctcggggatcctgtttgtgaatgacaccaaggccctgcggcggcccaagtgtgccgaacttcactacatggtggtggccaccgaccagcagacctctaggcaggcccaggcccagctgcttgtaacagtggaggggtcatatgtggccgaggaggcgggctgccccctgtcctgtgcagtcagcaagagacggctggagtgtgaggagtgtggcggcctgggctccccaacaggcaggtgtgagtggaggcaaggagatggcaaagggatcaccaggaacttctccacctgctctcccagcaccaagacctgccccgacggccactgcgatgttgtggagacccaagacatcaacatttgccctcaggactgcctccggggcagcattgttgggggacacgagcctggggagccccgggggattaaagctggctatggcacctgcaactgcttccctgaggaggagaagtgcttctgcgagcccgaagacatccaggatccactgtgcgacgagctgaggcgcgagttcctggttccgcgtggatccggaggcggtggaagcgacaaaactcacacatgcccaccgtgcccagcacctgaactcctggggggaccgtcagtcttcctcttccccccaaaacccaaggacaccctcatgatctcccggacccctgaggtcacatgcgtggtggtggacgtgagccacgaagaccctgaggtcaagttcaactggtacgtggacggcgtggaggtgcataatgccaagacaaagccgcgggaggagcagtacaacagcacgtaccgtgtggtcagcgtcctcaccgtcctgcaccaggactggctgaatggcaaggagtacaagtgcaaggtctccaacaaagccctcccagcccccatcgagaaaaccatctccaaagccaaagggcagccccgagaaccacaggtgtacaccctgcccccatcccgggatgagctgaccaagaaccaggtcagcctgacctgcctggtcaaaggcttctatcccagcgacatcgccgtggagtgggagagcaatgggcagccggagaacaactacaagaccacgcctcccgtgctggactccgacggctccttcttcctctacagcaagctcaccgtggacaagagcaggtggcagcaggggaacgtcttctcatgctccgtgatgcatgaggctctgcacaaccactacacgcagaagagcctctccctgtctccgggtaaaggcggccatcatcaccatcaccatcaccacgactacaaggacgacgatgacaagtag |

Table S3. Cryo-EM data collection parameters.

|  | **RET^WT^/GDF15/GFRAL** | **RET^C634R^/GDF15/GFRAL** |
| --- | --- | --- |
| Microscope (mode) | Titan Krios I (integrative) | Titan Krios II (counting) |
| Magnification | 75,000 | 130,000 |
| Voltage (kV) | 300 | 300 |
| Total electron dose (e^-^/Å^2^) | 60.3 | 62.88 |
| Exposure time (s) | 1.5 | 8 |
| Number of frames | 40 | 50 |
| Pixel size (Å) | 1.065 | 1.07 |

Movie S1. Conformational flexibility of the RET^WT^/GDF15/GFRAL complex revealed using models fitted in the cryo-EM densities. RET, GFRAL and GDF15 protomers are coloured in orange, blue and green, respectively.

Movie S2. Conformational flexibility of the RET^WT^/GDF15/GFRAL complex revealed by molecular dynamics simulation (front and top views). Two RET protomers are coloured in orange and yellow while GFRAL and GDF15 protomers are coloured in light grey and green, respectively. Calcium ions are shown in green spheres.

Movie S3. Conformational flexibility of the RET^C634R^ mutant dimer revealed by molecular dynamics simulation (front and top views). Two RET protomers are coloured orange and yellow. Calcium ions are shown in green spheres.
